# Qualitative differences in disease-associated MEK mutants reveal molecular signatures and aberrant signaling-crosstalk in cancer

**DOI:** 10.1038/s41467-022-31690-w

**Published:** 2022-07-13

**Authors:** Yuji Kubota, Yuko Fujioka, Ashwini Patil, Yusuke Takagi, Daisuke Matsubara, Masatomi Iijima, Isao Momose, Ryosuke Naka, Kenta Nakai, Nobuo N. Noda, Mutsuhiro Takekawa

**Affiliations:** 1 Division of Cell Signaling and Molecular Medicine, Institute of Medical Science, The University of Tokyo, Minato-ku, Tokyo, 108-8639 Japan; 2 Institute of Microbial Chemistry, Microbial Chemistry Research Foundation, Shinagawa-ku, Tokyo, Japan; 3 Division of Biological Molecular Mechanisms, Institute for Genetic Medicine, Hokkaido University, Sapporo, 060-0815 Japan; 4 Laboratory of Functional Analysis In Silico, Human Genome Center, Institute of Medical Science, The University of Tokyo, Minato-ku, Tokyo, 108-8639 Japan; 5 Molecular Pathology Laboratory, Institute of Medical Science, The University of Tokyo, Minato-ku, Tokyo, 108-8639 Japan; 6 Present Address: Combinatics Inc., Chiba, Japan

**Keywords:** Oncogenes, X-ray crystallography, Molecular medicine, Cancer therapy

## Abstract

Point-mutations of MEK1, a central component of ERK signaling, are present in cancer and RASopathies, but their precise biological effects remain obscure. Here, we report a mutant MEK1 structure that uncovers the mechanisms underlying abnormal activities of cancer- and RASopathy-associated MEK1 mutants. These two classes of MEK1 mutations differentially impact on spatiotemporal dynamics of ERK signaling, cellular transcriptional programs, gene expression profiles, and consequent biological outcomes. By making use of such distinct characteristics of the MEK1 mutants, we identified cancer- and RASopathy-signature genes that may serve as diagnostic markers or therapeutic targets for these diseases. In particular, two AKT-inhibitor molecules, PHLDA1 and 2, are simultaneously upregulated by oncogenic ERK signaling, and mediate cancer-specific ERK-AKT crosstalk. The combined expression of PHLDA1/2 is critical to confer resistance to ERK pathway-targeted therapeutics on cancer cells. Finally, we propose a therapeutic strategy to overcome this drug resistance. Our data provide vital insights into the etiology, diagnosis, and therapeutic strategy of cancers and RASopathies.

## Abstract (web summary)

MEK1 mutations are found in cancer and RASopathies, but their effects remain unclear. Here, the authors reveal a mutant MEK1 structure and qualitative differences in biological properties between the cancer- and RASopathy-associated mutants, providing insights into the pathophysiology, diagnosis, and treatment of these diseases.

## Introduction

The extracellular signal-regulated kinase (ERK) cascade, consisting of three tiers of protein kinases (Raf, MEK, and ERK), mediates mitogenic signals and is critical for a wide range of physiological and pathological processes, including cell proliferation, differentiation, embryonic development, and carcinogenesis^1^. In general, ERK signaling is initiated by growth factor [e.g., epidermal growth factor (EGF)] binding to its respective receptor tyrosine kinase (RTK), leading to Ras activation at the plasma membrane. Activated Ras interacts with and activates the Raf family protein kinases (ARaf, BRaf, and Raf-1), which then phosphorylate two conserved Ser residues within the T-loops of MEK1/2, which in turn phosphorylate ERK1/2. Activated ERK1/2 translocate from the cytoplasm to the nucleus where they phosphorylate and activate multiple substrates including several transcription factors (TFs) (e.g., Elk-1 and Sp1), resulting in the expression of the so-called immediate early genes (IEGs)^2^. Since many IEGs encode TFs (e.g., Egr1 and c-fos), their induction further modulates the expression of various target genes controlling a wide array of cellular responses. ERK1/2 are then gradually inactivated by dephosphorylation and return to the cytoplasm^3^. Several other mechanisms are also involved in the regulation of ERK signaling. These include scaffold proteins, docking interactions, protein phosphatases, and multiple feedback loops^1,4,5^. Proper regulation of such spatiotemporal dynamics of ERK signaling is critical for the maintenance of cellular functions and homeostasis.

Dysregulation of the ERK pathway is frequently observed in human cancers. Indeed, many key ERK pathway molecules are known oncogenes. Gain-of-function mutations or gene amplification of RTKs (e.g., EGFR), Ras, and BRaf are common in a variety of cancers^6^. Furthermore, MEK1 mutations, which are more frequent than those in MEK2, are also observed in many tumors such as melanoma, colorectal, lung, thyroid, and ovarian cancers^7^. These oncogenes ultimately hyperactivate ERK signaling and generate aberrant gene expression patterns that provoke cell overgrowth and/or a differentiation abnormality, thereby inducing cancer development and progression^8,9^. Targeting of these oncogenes for cancer therapy is therefore of particular interest, and their specific inhibitors (e.g., erlotinib, vemurafenib, and trametinib, inhibitors of EGFR, BRaf, and MEK, respectively) are currently in clinical use^10^. Although some of these ERK pathway-targeted drugs are clinically effective, their efficacy is limited by intrinsic and/or acquired resistance of cancer cells to these drugs. Furthermore, MEK mutations promote resistance to allosteric MEK inhibitors^11–13^. The precise molecular mechanisms underlying such drug resistance, however, remain ill-defined.

Besides their somatic mutations in sporadic cancers, recent clinical sequencing studies identified germline mutations of key components of ERK signaling (e.g., Ras, Raf, and MEK) in a group of congenital developmental disorders termed RASopathies^14^. RASopathies, including Noonan, Costello, Leopard, and cardio-facio-cutaneous (CFC) syndromes, share many overlapping clinical manifestations such as cranio-facial dysmorphisms, cardiomyopathies, neurocognitive impairment, and cutaneous and musculoskeletal abnormalities^15^. These congenital syndromes, except for CFC syndrome, are generally associated with an increased risk of malignancy^16^. However, the incidence of *MEK1* or *MEK2* gene mutation is higher in CFC syndrome (about 25% of all CFC cases) than in the other RASopathies^14^, suggesting that these germline MEK mutations are not necessarily related to cancer development. This finding implies that cancer- and RASopathy-associated MEK mutants may differentially alter ERK signaling processes and consequent biological outcomes. Although increased catalytic activity of the disease-associated MEK mutants has been reported^17^, the detailed biological properties of individual MEK mutants are, however, still obscure. Moreover, despite the importance of MEK1 in the etiology of cancer and RASopathies, no protein structure of any disease-associated MEK mutant is known.

Here, we report the crystal structure of a cancer-derived MEK1 mutant, and demonstrate the molecular mechanisms underlying abnormal kinase activities of the cancer- and RASopathy-associated MEK1 mutants. Furthermore, we show that differences in biochemical properties between cancer- and RASopathy-associated MEK1 mutants produce qualitative changes in ERK signaling dynamics and downstream transcriptional programs. Transcriptome analyses revealed that the two MEK1 mutant classes elicit distinct gene expression patterns that are specifically related to the pathophysiology of cancer or RASopathies. In particular, the AKT-inhibitor molecules PHLDA1 and PHLDA2 are simultaneously upregulated by oncogenic ERK signaling and confer resistance to ERK pathway-targeted therapeutics on cancer cells. Coadministration of bortezomib overcomes this drug resistance by disrupting PHLDA1/2-mediated, cancer-specific ERK-AKT crosstalk. Our data delineate qualitative differences in biological properties between cancer- and RASopathy-associated MEK1 mutants, and provide vital insights into the pathophysiology, diagnosis, and molecular basis for novel therapeutic strategies for these diseases.

## Results

### RASopathy- and cancer-associated MEK1 mutants display different biochemical and biological properties

**Fig. 1:**
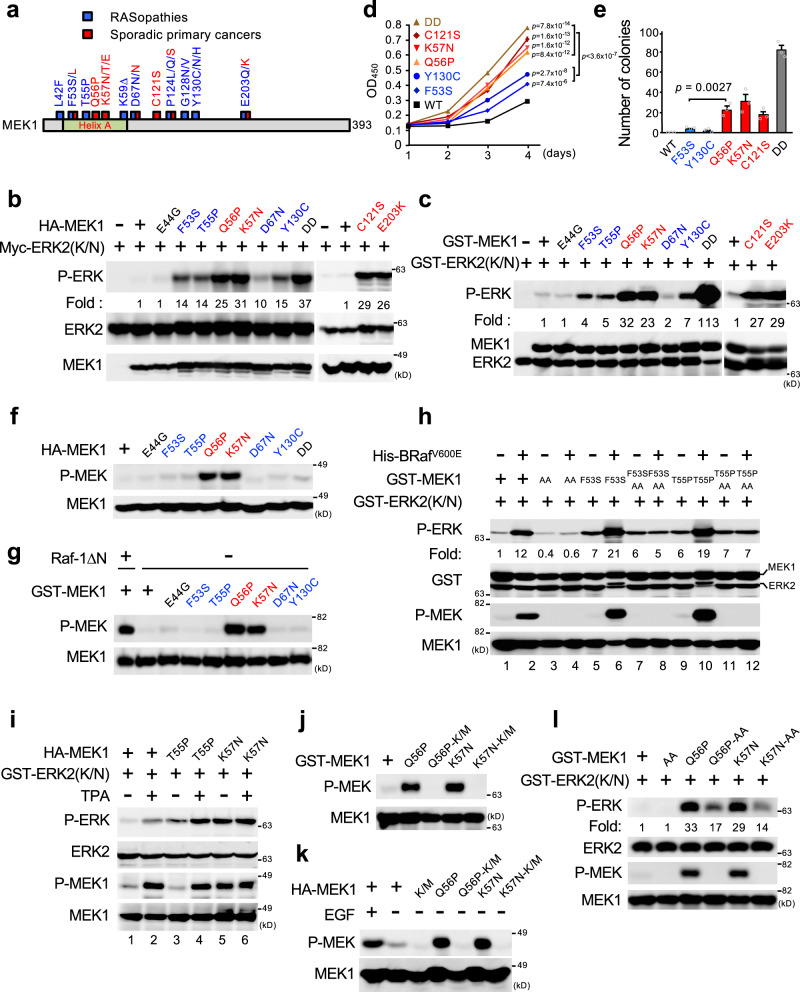
Rmuts and Cmuts exhibit different biochemical and biological properties. **a** Schematic diagram of MEK1 mutations in primary cancers (red) and RASopathies (blue). **b** HEK293 cells were transfected with wild type or mutated HA-MEK1 together with kinase-deficient Myc-ERK2(K/N) as indicated. Phosphorylated ERK (P-ERK) was detected by immunoblotting with an anti-phospho-ERK antibody (Ab) (top). Myc-ERK2(K/N) and HA-MEK1 expression levels are shown in the lower panels. MEK1(DD); a phosphomimetic, constitutively active mutant. **c** In vitro kinase assay of purified recombinant GST-MEK1 or its mutant derivatives using GST-ERK2(K/N) as a substrate. Phosphorylated ERK2(K/N) was detected by immunoblotting (top). Total GST-MEK1 and GST-ERK2(K/N) were also probed with an anti-GST Ab (bottom). **d**, **e** *MEK1*^−/−^ MEFs stably expressing HA-MEK1 or its indicated mutants were cultured in medium with 10% FBS (**d**) or were grown in soft agar for 3 weeks (**e**). In (**d**), cell proliferation was determined by CCK8 assay. OD, optical density. In (**e**) colonies larger than 0.1 mm were counted. **f**, **g**, **j**, **k** HA-MEK1 (WT or its indicated mutants) expressed in HEK293 cells (**f**, **k**) or bacterially expressed, purified GST-MEK1 (WT or its indicated mutants) (**g**, **j**) was immunoblotted with an anti-phospho-MEK1(S218/S222) Ab (P-MEK). Raf-1ΔN; N-terminally truncated, active Raf-1. K/M; a kinase-inactive K97M mutation. **h** Recombinant GST-MEK1 proteins were pre-incubated with (+) or without (−) active His-BRaf^V600E^, and then mixed with GST-ERK2(K/N). Phosphorylated ERK (P-ERK) and phosphorylated MEK1 (P-MEK) were detected by immunoblotting. AA; a non-phosphorylatable S218A/S222A mutant. **i** HEK293 cells were transfected with HA-MEK1 or its indicated mutants and were stimulated with (+) or without (−) TPA (for 20 min). Phosphorylated HA-MEK1 in cell lysates was monitored by immunoblotting (third). The kinase activity of immunoprecipitated HA-MEK1 was measured in an in vitro kinase assay using GST-ERK2(K/N) as a substrate as in **h**. **l** The indicated GST-MEK1 proteins were incubated with GST-ERK2(K/N), and the phosphorylation states of MEK1 and ERK2 were assessed by immunoblotting. **d**, **e** Data are mean ± SEM from three independent experiments. *P*-values were assessed using one-way ANOVA followed by Tukey’s multiple comparisons test (**d**) or using two-tailed Student t-test (**e**). Source data are provided as a Source Data file.

Recent genome-wide sequencing analyses of congenital RASopathies and primary sporadic cancers identified more than 20 point-mutations in the *MEK1* gene^17^ with different missense *MEK1* mutations in these two diseases (Fig. 1a). To explore if these MEK1 mutants display different, disease-specific, characteristics, we first investigated the kinase activities of individual MEK1 mutants in vivo. HEK293 cells were transiently transfected with HA-tagged wild-type (WT) MEK1, its single-nucleotide polymorphism (SNP) variant (E44G) that is found in healthy individuals^18^, its clinically-identified mutants found in RASopathies (hereafter referred to as Rmuts) (F53S, T55P, D67N, and Y130C)^18^ or in sporadic cancers (hereafter referred to as Cmuts) (Q56P, K57N, C121S, and E203K)^13^, or a constitutively active, phosphomimetic MEK1-S218D/S222D (DD) mutant, together with kinase-dead Myc-ERK2(K/N) as a substrate. The phosphorylation state of co-expressed Myc-ERK2(K/N) was then assessed by immunoblotting (Fig. 1b and Supplementary Fig. 1a). As expected, the kinase activity of MEK1(E44G) was similar to that of WT MEK1, whereas that of all the disease-associated MEK mutants was higher even under steady-state conditions, confirming that all the disease-associated MEK1 mutants tested are constitutively active. The activities of the Cmuts were, however, substantially higher than those of the Rmuts. Similarly, in an in vitro kinase assay using bacterially expressed, purified recombinant GST-MEK1 (WT or its derivative mutants), the Cmuts more efficiently phosphorylated GST-ERK2(K/N) in vitro than the Rmuts (Fig. 1c and Supplementary Fig. 1b–d). In contrast to their alteration of MEK kinase activity, none of the MEK1 mutations affected MEK1 cytoplasmic localization (Supplementary Fig. 1e). Thus, although all the disease-associated mutations rendered MEK1 constitutively active, the cancer-derived mutations conferred substantially higher kinase activity on MEK1 than the RASopathy-derived mutations in vivo and in vitro.

Next, we examined if these MEK1 mutations affected cellular processes for which ERK signaling is crucial, such as proliferation and cell transformation. Stable expression of one of the MEK1 mutants (Q56P, K57N, C121S, F53S, or Y130C) in *MEK1*^−/−^ mouse embryonic fibroblasts (MEFs) significantly enhanced cell proliferation, compared to the WT counterpart (Fig. 1d). Consistent with their higher kinase activities, the proliferation of cells expressing a Cmut (Q56P, K57N, or C121S) was significantly greater than that of cells expressing a Rmut (F53S or Y130C). Moreover, a soft-agar colony formation assay using *MEK1*^−/−^ MEFs showed that the Cmuts, but not the Rmuts, induced anchorage-independent growth, a hallmark of oncogenic transformation (Fig. 1e). Therefore, the high kinase activities of the Cmuts are required for efficient induction of transformation. Thus, although all of the disease-associated MEK1 mutants are constitutively active, the somatic MEK1 mutants found in sporadic primary cancers are highly catalytically active and efficiently induce oncogenic transformation, whereas the germline MEK1 mutants detected in RASopathies are relatively moderately active and are therefore essentially non-oncogenic.

### Cancer-derived MEK1 mutants acquire the ability to undergo autophosphorylation

We next investigated the detailed molecular mechanisms underlying the higher kinase activities of the Cmuts vs. the Rmuts. Since MEK is primarily activated by Raf-mediated phosphorylation at the S218 and S222 residues in its T-loop, we initially assessed the S218/S222 phosphorylation states of the HA-tagged MEK1 mutants expressed in HEK293 cells by immunoblotting. In the absence of growth factor stimulation (and therefore without Raf activation), like WT MEK1, no phosphorylation was detected in the Rmuts, despite their elevated kinase activities (Fig. 1f and Supplementary Fig. 1f). In contrast, the Cmuts (Q56P, K57N, C121S, and E203K) were markedly phosphorylated even under steady-state conditions, consistent with their high kinase activity (Fig. 1f and Supplementary Fig. 1g, h). Furthermore, immunoblot analysis of the bacterially produced recombinant MEK mutants also showed that the Cmuts, but not the Rmuts, were phosphorylated even when expressed in E. coli (Fig. 1g and Supplementary Fig. 1i). Thus, the RASopathy-derived mutations constitutively, but moderately, activate MEK1 in a manner independent of T-loop phosphorylation, whereas the cancer-derived mutations markedly augment MEK1 activity through the T-loop phosphorylation by a Raf-independent mechanism.

Next, to further verify the T-loop-phosphorylation-independent activation of the Rmuts, we mutated the activating phosphorylation sites (S218A/S222A; AA) in the T-loops of the mutants and assessed their kinase activities in an in vitro kinase assay. Initially, we confirmed that, while GST-MEK1(F53S) and (T55P) were efficiently phosphorylated by active BRaf^V600E^ in vitro and this phosphorylation further augmented their kinase activities (Fig. 1h, lanes 6 and 10), MEK1(F53S)-AA, and (T55P)-AA were refractory to the BRaf^V600E^-induced T-loop phosphorylation and activation (lanes 8 and 12). However, interestingly, MEK1(F53S)-AA, and (T55P)-AA retained the increased basal kinase activities, which are almost equal to those of their counterparts without AA mutations (compare lanes 5 and 7; and lanes 9 and 11). Similarly, MEK1(F53S)-AA expressed in cells exhibited a higher basal kinase activity than WT MEK1 (Supplementary Fig. 1j). Thus, the RASopathy-derived mutations moderately, but constitutively, increase basal MEK1 activity independently of T-loop phosphorylation, but they can be further activated by Raf-mediated phosphorylation. Indeed, MEK1(T55P) kinase activity was further elevated when Raf was activated by 12-O-tetradecanoylphorbol 13-acetate (TPA) treatment, as assessed by an in vitro kinase assay (Fig. 1i, lane 4).

Since a recent study has reported that some MEK1 mutants can autophosphorylate their own T-loops^17^, we next explored if the observed T-loop phosphorylation of Cmuts resulted from their abnormal autophosphorylation activity. To test this idea, we additionally introduced a kinase-inactivating mutation (K97M; K/M) into several Cmuts (Q56P, K57N, C121S, and E203K), and assessed their phosphorylation states by immunoblotting. Introduction of the K/M mutation completely abrogated their T-loop phosphorylation when the proteins were expressed in *E. coli* (Fig. 1j and Supplementary Fig. 1i, k) and in HEK293 cells (Fig. 1k and Supplementary Fig. 1h). Thus, all the Cmuts tested acquired the ability to autophosphorylate their own T-loops, thereby inducing strong kinase activities. Furthermore, we also found that, as was the case for the Rmuts, even in the absence of T-loop phosphorylation, MEK1(Q56P)-AA, (K57N)-AA, and (C121S)-AA mutants still exhibited moderately elevated basal kinase activities vs. MEK1 WT (Fig. 1l and Supplementary Fig. 1l). Thus, the strong kinase activities of the Cmuts are generated by a combination of two distinct (T-loop phosphorylation-independent and -dependent) mechanisms: Cmuts are moderately active even without T-loop phosphorylation, but they also acquire the ability to autophosphorylate their own T-loops, thereby possessing strong activities sufficient for cell transformation. The auto-phosphorylation occurs intra-molecularly, as these mutants did not phosphorylate exogenous MEK1 or MEK2 proteins (Supplementary Fig. 1m–o).

### Disease-associated MEK1 mutations confer resistance to ERK pathway-targeted therapeutics

Several MEK1 mutations have been detected not only in primary (pre-treatment) cancers but also in recurrent cancers in patients treated with ERK pathway-targeted drugs (e.g., EGFR, Raf, or MEK inhibitors) (Supplementary Fig. 2a). Some of these mutations are shown to develop after treatment initiation and are associated with acquired resistance of tumor cells to the anti-cancer drugs^12,13^. These treatment-induced MEK1 mutations include those detected not only in primary cancers (F53L, C121S, and E203K) but also in RASopathies (P124Q and P124L), suggesting that any of the disease-associated MEK1 mutants can confer resistance to ERK pathway-targeted therapeutics on tumor cells. To test this, we stably expressed disease-representative MEK mutants (cancer-derived Q56P, K57N, and C121S; RASopathy-derived F53S and Y130C) in A375 melanoma cells harboring BRaf^V600E^ (Supplementary Fig. 2b), and assessed their effects on the sensitivity of the tumor cells to BRaf- or MEK-inhibitors. All of the A375 cells expressing a MEK1 mutant showed higher proliferation than control cells even in the presence of high concentrations of the BRaf inhibitor (vemurafenib) (Supplementary Fig. 2c, d). Similarly, expression of any of the MEK mutants conferred substantial resistance to a MEK inhibitor (selumetinib) on the tumor cells, with concentrations required to achieve 50% growth inhibition (GI50) being more than 10-fold higher than that observed in control cells (Supplementary Fig. 2e, f). Moreover, A375 cells expressing MEK1(C121S) were highly resistant to various allosteric MEK inhibitors (Supplementary Fig. 2g, h). Thus, any of the disease-associated mutations sufficed to develop resistance of cancer cells to the ERK pathway-targeted drugs, explaining why both classes of MEK1 mutations are detected in recurrent cancers. Furthermore, since all the mutants tested were resistant to the allosteric MEK inhibitors, these mutations may similarly provoke MEK1 structural distortion that interferes with inhibitor binding.

### Structural basis for an activating MEK mutation

**Fig. 2:**
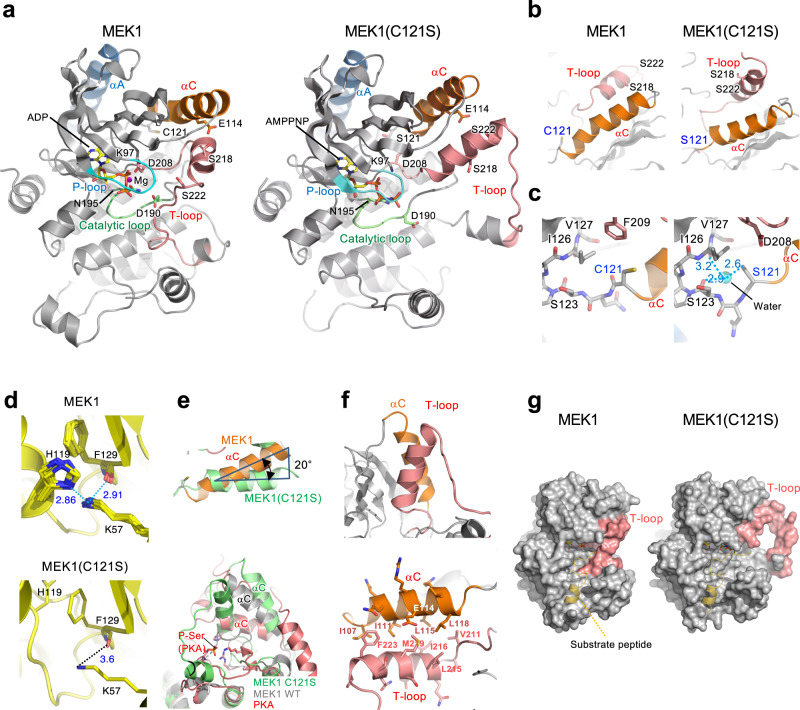
Structural basis for an activating MEK mutation. **a** Overall crystal structures of MEK1 (PDB:3EQI) and MEK1(C121S) are shown as ribbon diagrams. Color coding: dark blue, helix A (αA); orange, helix C (αC); pink, T-loop; cyan, P-loop. The positions of key residues are shown. **b** Close-up views of the helix C and T-loop in MEK1 and MEK1(C121S). **c** Local structures around WT C121 and the mutated S121. Cyan sphere, water molecule; light-blue dotted lines, H-bonds; numbers, distances in (Å). **d** MEK1(C121S) mutation disrupts the interdomain hydrogen (H)-bonds connecting residues H119 and F129 with K57. Superposition of the interdomain H-bonds in 14 MEK1 structures (upper) and the corresponding area in MEK1(C121S) (lower) are shown. **c**, **d** Nitrogen and oxygen atoms are colored blue and red, respectively. The numbers beside the dotted lines represent distances (Å). **e** Excessive outward rotation of the MEK1(C121S)-helix C. (Upper) The helix C structure of MEK1(C121S) (green) is superimposed on that of MEK1 WT (orange, PDB: 3SLS). The helix C of MEK1(C121S) is rotated out by 20 degrees relative to that of MEK1 WT. (Lower) Superposition of the C helices of MEK1(C121S) (green), MEK1 WT (grey, PDB: 3SLS), and active PKA (red, PDB: 1ATP). **f** Antiparallel coiled-coil interactions between the helix C and the T-loop in MEK1(C121S). (upper) The helix C interacts with a part of the T-loop that forms a helix. (lower) Residues forming hydrophobic contacts are shown. **g** Space-filling models. A PKA substrate peptide (yellow, PDB: 4HPU) is superimposed on the MEK1 structures.

Previous structural analyses of inactive MEK1 suggested that its helix A, which is present in the N-terminal extension of the kinase domain and is a structural feature unique to the MEK family kinases, stabilizes the inactive conformation of the N-terminal lobe of the kinase^19,20^ (Supplementary Fig. 3a). These data implied that the disease-associated MEK1 mutations were likely to compromise the structural integrity of the inhibitory helix A. To date, however, the crystal structure of a MEK mutant or of activated WT MEK has not yet been determined. To gain insight into the structural basis of the active MEK mutants, we sought to elucidate the conformational alteration of the Cmuts. Among several mutants tested, we succeeded in crystallizing MEK1(C121S) and determined its X-ray structure at 2.0 Å resolution (Fig. 2a). The WT C121 residue is in the N-lobe proximal to the helix C (P105-H119), which generally serves as a regulatory element of protein kinases^19^ (Fig. 2b), and resides juxtaposed to the helix A (Supplementary Fig. 3a). Although MEK1(C121S) retained the bilobal architecture typical of protein kinases, it had several structural features that were unique to the mutant.

Firstly, C121S mutation not only led to destabilization of the inhibitory helix A, but also affected the orientation and conformation of the helix C and the T-loop. In the mutant, the relatively small side chain of the mutated S121 residue faces in the opposite direction relative to the larger side chain of the WT C121 and forms a hydrogen (H)-bond with a water molecule (Fig. 2c and Supplementary Fig. 3b). This water molecule further creates two H-bonds with the main-chain carbonyl oxygens of S123 and I126. These water-bridged, indirect interactions between S121, S123, and I126 stabilize and fix the local structure around S121 and affect the conformation of the adjacent helix C. In contrast to inactive WT MEK1, whose helix C is a hybrid of N-terminal α-helix (residues 105–115) and C-terminal 3_10_-helix (residues 116–120) with a kink between them, the helix C of MEK1(C121S) (residues 107–119) is entirely α-helical without a kink, becoming ~7 Å shorter than that of WT, and is displaced from its original position (Fig. 2b and Supplementary Fig. 3c). These conformational alterations move H119 and F129 residues (on the helix C and β4 strand, respectively) away from K57 on the helix A and disrupt the H-bonds connecting H119 and F129 with K57, which are observed in WT MEK1 (Fig. 2d and Supplementary Fig. 3a). These changes destabilize the inhibitory helix A, as confirmed by its higher crystallographic B factor in MEK1(C121S) than that in WT MEK1 (Supplementary Fig. 3d). Since these H-bonds fix the inhibitory helix A to the helix C, previous in silico simulation studies assumed that disruption of this interaction by mutation would provoke an inward rotation of the freed helix C in order to form a salt-bridge between E114 (on the helix C) and the catalytic K97 (on the β3-strand), and to complete the so-called αC-in conformation, which is, in general, a structural feature of active kinases^21^. Interestingly, however, we detected neither the salt-bridge nor the αC-in conformation in MEK1(C121S), despite its strong kinase activity (Fig. 2a and Supplementary Fig. 3e). Contrary to the previous assumption, the MEK1(C121S)-helix C does not move inward, but rather, it further rotates out from the N-lobe at an angle of 20° relative to that of WT MEK1 (Fig. 2e). This excessive outward displacement allows the helix C to create a coiled-coil-mediated stable interaction with the newly-formed α helix (residues 213–224) in the T-loop (Fig. 2f), thereby inducing an extended, open conformation of the T-loop (Fig. 2a, g, and Supplementary Fig. 3f, g). This conformational change favors substrate binding. In contrast to inactive MEK1, in which the T-loop is in a closed conformation and partially conceals the substrate binding region as well as the active-site cleft of the kinase (Fig. 2g), the extended T-loop structure of MEK1(C121S) uncovers these regions, thereby rendering this mutant readily accessible to its substrate. Therefore, a series of these structural alterations at least partly explain the constitutive kinase activity of MEK1(C121S).

Secondly, we found that disruption of the H-bonds of K57 to H119 and F129 is critical for acquisition of the abnormal autophosphorylating activity of the Cmuts. Disruption of the H-bonds by point-mutations at any of these residues, i.e., H119Y, F129L, or/and K57N (all of which are indeed detected in cancer cell lines or clinical cancers)^17^, sufficed to allow MEK1 to undergo autophosphorylation (Supplementary Fig. 4a–d). Moreover, substitution of Q56 in the helix A only by a helix-disrupting Pro (which may severely alter the orientation of its adjoining K57, and is indeed detected in cancer), but not by other amino-acids, conferred autophosphorylating capability on MEK1 (Supplementary Fig. 4c, d). Therefore, these findings indicate that only mutations that lead to severe distortion of the local structure around the H-bonds can elicit the MEK1 autophosphorylation.

**Fig. 3:**
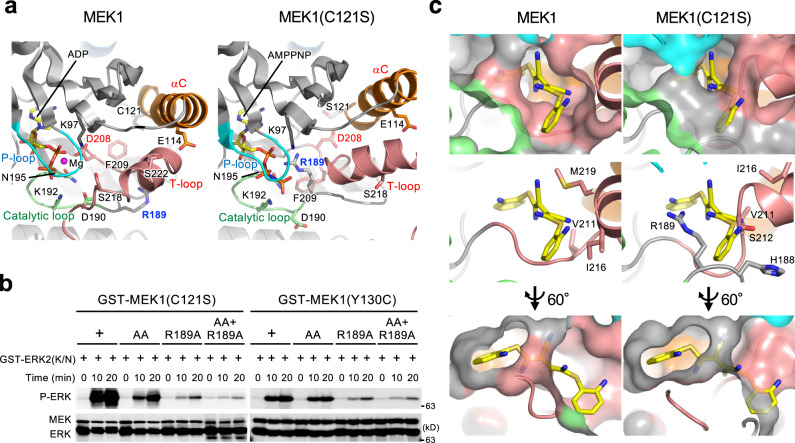
MEK1(C121S) exhibits a unique ATP-binding property and deformity of the allosteric pocket. **a**, **b** R189 mediates ATP-binding in MEK1 mutants. **a** Close-up views around R189. The R189 side-chain in MEK1(C121S) is rotated towards the ATP-binding site and directly coordinates an ATP analog, AMPPNP. **b** In vitro kinase assay of recombinant proteins. The indicated MEK1 mutants were incubated with GST-ERK2(K/N). ERK phosphorylation was probed with an anti-P-ERK Ab. Source data are provided as a Source Data file. **c** Close-up views of the MEK1 allosteric pockets. The MEK inhibitor U0126 in the MEK1-U0126 complex (PDB: 3EQH) (left) is superimposed onto MEK1(C121S) (right).

Thirdly, the MEK1(C121S) mutant exhibited a unique ATP-binding property. Previous studies showed that many active kinases and some inactive kinases (including MEK1) adopt the DFG-in conformation, in which the D side-chain of the conserved DFG motif at the N-terminus of the T-loop points into the ATP-binding site and coordinates an ATP-bound Mg^2+^ ion (Mg-ATP) to facilitate catalysis (Fig. 3a, left)^21^. In contrast, the DFG motif of MEK1(C121S) adopted the DFG-out conformation. The D208 side chain flips away from the ATP-binding site and thus fails to bind Mg-ATP (Fig. 3a, right). Instead of D208-ATP binding, R189 (in the catalytic loop HRD motif) mediates ATP-binding in MEK1(C121S). The R189 side chain is rotated toward the ATP-binding site and directly coordinates AMPPNP (an ATP analog) via the formation of electrostatic interactions with the phosphate groups of AMPPNP. Since substitution of Ala for R189 (R189A) profoundly curtailed the kinase activities of this and other (K57N and Y130C) MEK mutants (Fig. 3b and Supplementary Fig. 4e, f), this R189-mediated, atypical ATP-binding is likely to be critical for their enhanced kinase activities.

Finally, C121S mutation perturbs the local structure of the allosteric pocket where therapeutic MEK inhibitors bind. Allosteric MEK inhibitors are known to interact with specific residues, including V211 and S212, at the rim of the pocket in WT MEK1 (Fig. 3c)^22^. These residues are, however, displaced from their original positions in MEK1(C121S), leading to deformation and reduction of the pocket space. The superimposed model of MEK1(C121S) and U0126 showed that U0126 failed to dock into the deformed pocket (Fig. 3c, right). Therefore, this mutation confers resistance to its therapeutic inhibitors on MEK1 by evoking deformity of the allosteric pocket. In contrast, the ATP-binding pocket remained intact. Consistent with these data, we found that the ATP-competitive MEK inhibitor, hymenialdisine^23^, can more potently inhibit MEK1(C121S) than WT MEK1 (Supplementary Fig. 4g, h).

### The two classes of MEK mutations differentially affect the spatiotemporal dynamics of ERK signaling

**Fig. 4:**
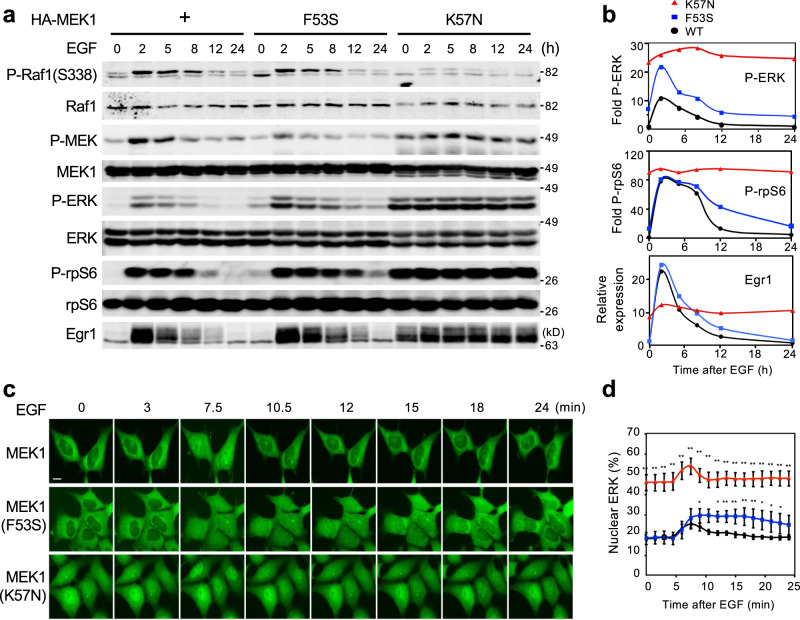
MEK mutations alter the spatiotemporal dynamics of ERK signaling. **a**, **b** Disease-associated MEK mutants differentially affect ERK signaling. **a** HEK293 cells stably expressing HA-MEK1 WT ( + ), F53S, or K57N were treated with EGF for the indicated times. The phosphorylation levels of Raf1, MEK1, ERK1/2, and rpS6 were analyzed by immunoblotting using appropriate phospho-specific Abs. The expression levels of Raf1, MEK1, ERK1/2, rpS6, and Egr1 in cell lysates are also shown. **b** The intensity of the P-ERK1/2 (top), P-rpS6 (middle), and Egr1 (bottom) bands in **a** was quantified. **c**, **d** The indicated cells stably expressing ERK1-GFP were treated with EGF for the indicated times. **c** ERK1 localization was visualized by GFP fluorescence. Scale bar, 10 μm. **d** Time course of the changes in the nuclear localization of ERK following EGF treatment. The percentage of nuclear fluorescence relative to that in the whole cell was calculated. Data are mean ± SEM from five independent experiments. *P*-values were determined using one-way ANOVA followed by Tukey’s multiple comparisons test. *p < 0.05; **p < 0.01. Source data are provided as a Source Data file.

To examine if the two classes of the MEK mutations differentially affect ERK signaling processes, we established HEK293 cells stably expressing HA-MEK1, or a representative disease-associated MEK mutant (RASopathies, F53S; Cancer, K57N) (Supplementary Fig. 5a). We then stimulated these cells with EGF, and monitored the phosphorylation states of key molecules of ERK signaling [Raf-1, MEK1, ERK, and ribosomal protein S6 (rpS6)] and ERK-mediated expression level of an IEG, Egr1, by immunoblotting (Fig. 4a, b and Supplementary Fig. 5b–d). In the WT MEK1 control cells, the phosphorylation levels of Raf-1, MEK1, ERK, and rpS6 peaked within 2 h after EGF stimulation, and then gradually returned to the basal levels by 24 h. Accordingly, Egr1 protein expression was transiently induced with a time course similar to that observed for ERK activity. However, in MEK1(F53S)-expressing cells (hereafter called F53S cells), consistent with the moderately elevated basal MEK1 activity, the phosphorylation levels of its downstream molecules ERK and rpS6 were moderately increased vs. WT MEK1 control cells even without EGF (at time 0). EGF stimulation of F53S cells led to a more prolonged and exaggerated increase in ERK and rpS6 phosphorylation and in Egr1 expression, compared with control cells. Moreover, in cells expressing the cancer-derived K57N mutant (termed K57N cells), MEK1(K57N), ERK, and rpS6 were persistently phosphorylated and therefore Egr1 was constitutively expressed irrespective of EGF stimulation. These results reflected the K57N mutant autophosphorylation and its consequent high kinase activity. In contrast, activating phosphorylation of Raf-1 at S338 was profoundly suppressed in K57N cells, most likely due to its negative feedback regulation by activated ERK^24^. Essentially identical results were also obtained for another Rmut (Y130C) and Cmut (Q56P) (Supplementary Fig. 5e).

Next, to assess the effects of the MEK mutations on the spatiotemporal dynamics of the ERK molecule, we introduced GFP-tagged ERK1 into the cell lines and monitored its nuclear translocation in real time by fluorescence microscopy (Fig. 4c, d and Supplementary Movie 1–3). In line with previous reports^25^, following EGF stimulation, the control cells exhibited rapid and transient nuclear import of ERK. In contrast, in F53S cells, ERK was more efficiently translocated into the nucleus, and the duration of its nuclear localization was markedly prolonged. In K57N cells, a considerable cohort of ERK was constitutively localized in the nucleus even without EGF, and EGF treatment transiently augmented its nuclear localization. These findings therefore indicated that the Rmut not only moderately enhanced basal ERK activity but also considerably increased the magnitude and duration of growth factor-induced ERK signaling, and led to the prolonged nuclear accumulation of ERK. In contrast, the Cmut elicited persistent activation and nuclear accumulation of ERK irrespective of growth factor stimulation. Thus, the two classes of MEK mutants differentially altered the spatio-temporal dynamics of ERK signaling and the expression pattern of IEGs such as Egr1.

### MEK mutations generate distinct gene expression patterns

**Fig. 5:**
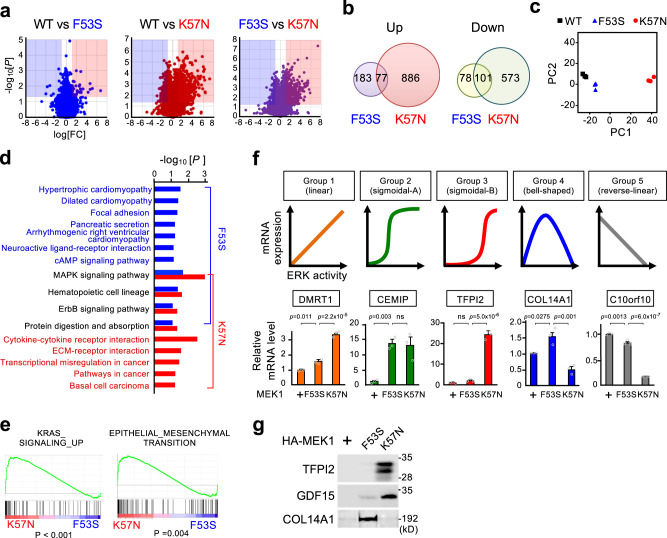
MEK mutations generate distinct gene expression patterns. **a** Transcriptome analyses of HEK293 cells stably expressing HA-MEK1 WT, F53S, or K57N. Genes differentially expressed between WT and F53S (left), WT and K57N (middle), and F53S and K57 (right) are displayed as volcano plots (*x*-axis: fold-change [log2], *y*-axis: *p*-value [-log10]). Red area, log_2_FC > 1 and *p* < 0.05 (*n* = 3); blue area, log_2_ FC < −1 and *p* < 0.05 (*n* = 3). **b** Venn diagrams illustrating the number of genes significantly upregulated (>2-fold; left) and downregulated (<0.5-fold; right) in F53S and K57N cells vs. WT cells. **c** Principal component analysis of the gene expression profiles of WT, F53S, and K57N cells. **d** KEGG pathway enrichment analyses of genes upregulated in F53S and K57N cells vs. WT cells. **e** Gene sets enriched analysis. The indicated gene signatures were significantly enriched in K57N cells compared with F53S cells. **f** Five distinct patterns of gene expression relative to the degree of aberrant ERK activity in F53S and K57N cells. (Upper) Schematic representations of the gene expression patterns. (Lower) qRT-PCR analysis of the expression level of a representative gene in each group. Data are mean ± SEM from three independent experiments. *P*-values were determined using one-way ANOVA followed by Tukey’s multiple comparisons test. ns, not significant. **g** Immunoblot analysis of representative secreted proteins of the sigmoidal-B group (TFPI2 and GDF15) and the bell-shaped group (COL14A1) in cell culture supernatants from the indicated cells. Source data are provided as a Source Data file.

Since many IEGs encode TFs, the distinctive patterns of IEG expression in F53S and K57N cells vs. WT control cells imply that global gene expression profiles would also be differentially influenced by these two mutations. To test this idea, we performed a genome-wide transcriptome analysis using the 60 K SurePrint G3 Human gene expression microarray technology. Total RNA was extracted from the three cell lines under steady-state conditions and was used for analysis of their gene expression profiles. Based on three independent experiments in each cell line, we identified that F53S and K57N mutations significantly upregulated a total of 260 and 963 genes, respectively (adjusted *p* < 0.05, >2-fold increase vs. control cells), of which there were only 77 overlapping genes (Fig. 5a, b). Furthermore, a total of 179 and 674 genes were downregulated in F53S and K57N cells, respectively (adjusted *p* < 0.05, <0.5-fold decrease), and only 101 of these genes were commonly decreased. Therefore, although these two mutations up- or down-regulate some genes in common, each mutation can alter the expression of a larger number of unique genes. Consistent with these data, principal component analysis (PCA) revealed that these three cell lines segregated each other in their gene expression profiles, with K57N cells showing a greater separation from control cells than F53S cells (Fig. 5c). Moreover, KEGG pathway analyses of the MEK1(F53S)- and (K57N)-induced differentially expressed genes (DEGs) showed that only a few biological pathways (e.g., MAPK and ErbB signaling pathways) were affected in common by these two mutations, but instead, each mutation impacted on a unique set of pathways (Fig. 5d). Consistent with the fact that cardiomyopathy and psychomotor retardation are the most frequent clinical features of the RASopathies^15^, the F53S-induced DEGs were enriched in pathways that are involved in various types of cardiomyopathies and neuroactive ligand-receptor interaction. In contrast, the K57-induced DEGs were significantly enriched in the pathways related to cancer (e.g., transcriptional misregulation in cancer, and pathways in cancer). Gene set enrichment analysis (GSEA) also demonstrated that K57N cells, but not F53S cells, were enriched for genes involved in active K-Ras signaling and in epithelial-mesenchymal transition, a characteristic feature of invasive cancer cells (Fig. 5e). Therefore, MEK1(F53S) and (K57N) mutations alter the expression of different sets of genes that are involved in the pathogeneses of the RASopathies and cancers, respectively.

Interestingly, based on these surveys, we also found that alterations in gene expression in F53S and K57N cells relative to the degree of aberrant ERK activity could be classified into at least five distinct patterns (Groups 1–5) (Fig. 5f and Supplementary Fig. 6a): (1) linear increase, where the level of gene expression increased in proportion to ERK activity. In this pattern, as expected, gene induction is more efficient in K57N cells than in K53S cells; (2) sigmoidal-A, where the gene expression was enhanced vs. control to the same extent in F53S and K57N cells with increasing ERK activity; (3) sigmoidal-B, where gene expression was strongly enhanced only under conditions of high ERK activity. Therefore, such gene induction is specific to K57N cells; (4) bell-shaped, where gene expression was enhanced only under conditions of moderate ERK activity. Such gene induction is specific to F53S cells; (5) linear decrease, where gene expression decreased in inverse proportion to ERK activity. The expression levels of the representative genes in the individual patterns were validated using qRT-PCR (Fig. 5f, lower graphs). We confirmed that these five expression patterns were generated depending on the degree of cellular ERK activity but not on the particular type of MEK1 mutation, as these patterns were reproduced when cellular ERK activity was gradually increased by drug-induced expression of the MEK1(DD) mutant using the ProteoTuner-inducible expression system (Supplementary Fig. 6b, c).

Since the genes of the sigmoidal-B group and of the bell-shaped group are expressed preferentially in K57N and F53S cells, respectively, we next tested if the proteins encoded by these genes are also specifically upregulated in the corresponding cells (Fig. 5g and Supplementary Fig. 6d). Western blotting showed that the TFPI2 and COL14A1 proteins, which are secreted proteins of the sigmoidal B and the bell-shaped group, respectively, were indeed only detected in the cell culture supernatant of K57N and F53S cells, respectively. This result suggested that TFPI2 and COL14A1 may serve as serum (or amniotic fluid) diagnostic markers specific for cancer (TFPI2) and RASopathies (COL14A1). Consistent with our data, serum TFPI2 levels were reported to be significantly elevated in patients with ovarian cancer^26^. These combined findings indicate that the RASopathy- and the cancer-associated MEK1 mutations generate distinct spatio-temporal properties of ERK signaling and thereby differentially alter the global gene expression profiles in cells. Such effects may account for the distinct clinical manifestations (developmental abnormality vs. carcinogenesis) of these two diseases even though the same *MEK1* gene is mutated in both diseases (Supplementary Fig. 6e).

### Genes upregulated by oncogenic MEK1(K57N) serve as cancer signature genes

**Fig. 6:**
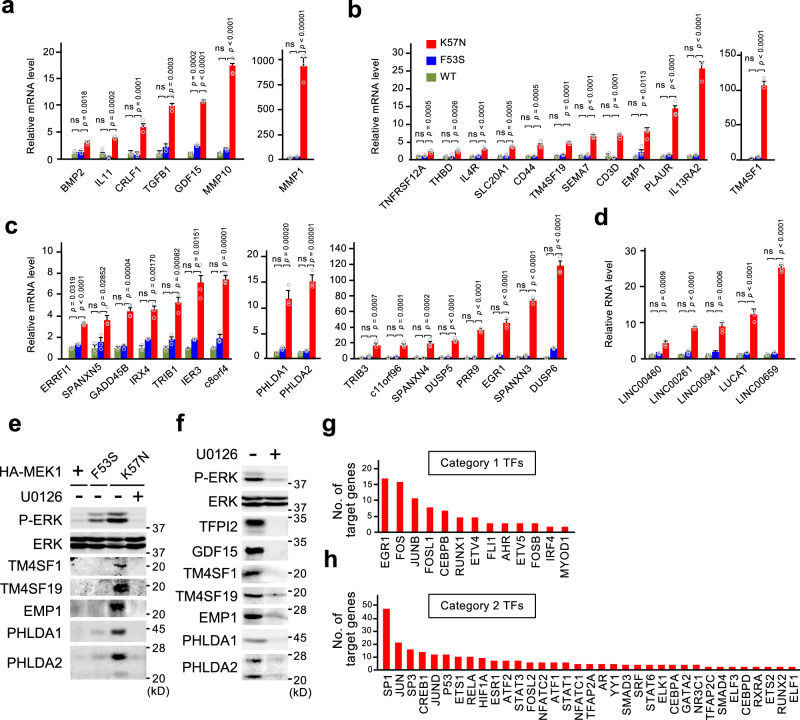
Genes upregulated by oncogenic MEK1(K57N) serve as cancer signature genes. **a**–**d** qRT-PCR analyses of the sigmoidal-B group of genes. The genes were classified as encoding secreted (**a**), transmembrane (**b**), or cytoplasmic/nuclear (**c**) proteins, or long intergenic non-coding RNAs (lncRNAs) (**d**). Data are mean ± SEM from three independent experiments. *P*-values were assessed using one-way ANOVA followed by Tukey’s multiple comparisons test. ns, not significant. **e**, **f** The expression levels of the indicated proteins in HEK293 cells expressing HA-MEK1(WT, F53S, or K57N) (**e**) and in H1299 cells (**f**) were assessed by immunoblotting with the appropriate Abs. Where indicated, the cells were pretreated with the MEK inhibitor U0126 for 24 h. **g**, **h** TransFac analysis of the promoter regions of the genes upregulated (>2-fold, *p* < 0.05) in K57N cells. The graphs show the number of target genes of the transcription factors (TFs) that are increased (>2-fold) in expression (**g**) and of the TFs that show no or weak change (0.5- to 2-fold) in expression (**h**) vs. control cells. Source data are provided as a Source Data file.

An interesting corollary of the above findings is that genes whose expression is upregulated only by the oncogenic MEK1(K57N) mutant (i.e., the sigmoidal-B group) can serve as cancer signature genes, which might serve as cancer diagnostic markers or therapeutic targets. Using qRT-PCR analysis, we validated at least 42 genes that met the criteria of the sigmoidal-B group. These genes encode 8 secreted proteins, 12 trans-membrane proteins, 17 cytoplasmic and/or nuclear proteins, and 5 long non-coding RNAs (lncRNAs) (Fig. 6a–d and Supplementary Fig. 6f). Indeed, analyses of large-scale cancer genomics datasets showed that many of these genes were upregulated in various cancer tissues compared with their corresponding normal tissues (Supplementary Fig. 7a–c). This group of genes included known cancer signature genes that encode tumor-associated antigens (e.g., TFPI2, MMP1, and CD44) and cancer-testis antigens (e.g., IL13RA2, and SPANXN3/4/5)^27^. More important, this group of genes also included genes whose roles in cancer have yet to be fully addressed (e.g., TM4SF1, TM4SF19, EMP1, PHLDA1, and PHLDA2). We confirmed that the latter five genes were upregulated at the level of protein in K57N, but not in F53S or WT cells (Fig. 6e and Supplementary Fig. 7d), and in H1299 cancer cells harboring NRas^Q61K^ in which ERK signaling is hyperactivated (Fig. 6f). We showed that their expression was abrogated by cell treatment with MEK inhibitors (U0126 or trametinib), indicating their ERK-dependent expression (Fig. 6e, f and Supplementary Fig. 7e, f). Thus, these genes are induced by oncogenic ERK activity and may serve as molecular signatures of cancers with hyperactive ERK signaling.

Next, to analyze TFs responsible for the altered gene expression in cells with oncogenic ERK activity, we performed transcription factor binding site (TFBS) enrichment analysis of the promoter regions of the genes whose expression is significantly upregulated (>2-fold vs. control, *p* < 0.05) in K57N cells [K57N-upregulated genes (UGs)] using the TransFac database, which contains experimentally-proven TFBSs and regulated genes^28^. Since the expression levels of some TFs themselves were upregulated in K57N cells, we classified the TFs correlated with the enriched TFBSs into two categories: (1) TFs whose expression is increased (>2-fold), and (2) TFs that show no or weak change in expression (0.5- to 2-fold) in K57N cells. TFBSs of specific TFs in each category were significantly enriched in K57N-UGs. In category 1, as anticipated, most of the TFs correlating with the identified, enriched TFBSs are members of the IEGs such as Egr1, FOS, JUNB, and FOSL1 (Fig. 6g). In category 2, the enriched TFBSs are sites for TFs that are directly (e.g., SP1, SP3, JUN, JUND, and ETS1) or indirectly (e.g., CREB1) phosphorylated and activated by ERK (Fig. 6h). The TFs that best correlated with the enriched TFBSs in category 2 were TFs of the SP and JUN family members, and many (more than a dozen) target genes of individual TFs of these families are upregulated in K57N cells. However, K57N-UGs showed little or no enrichment for TFBSs of two prominent ERK-substrate TFs, ELK1 (only 4 sites) or Myc (no site). Thus, these findings suggest that oncogenic ERK signaling selectively activates specific TFs to generate the cancer-specific gene expression pattern.

### PHLDA1/2 are highly expressed in cancers with ERK-activating oncogenes

**Fig. 7:**
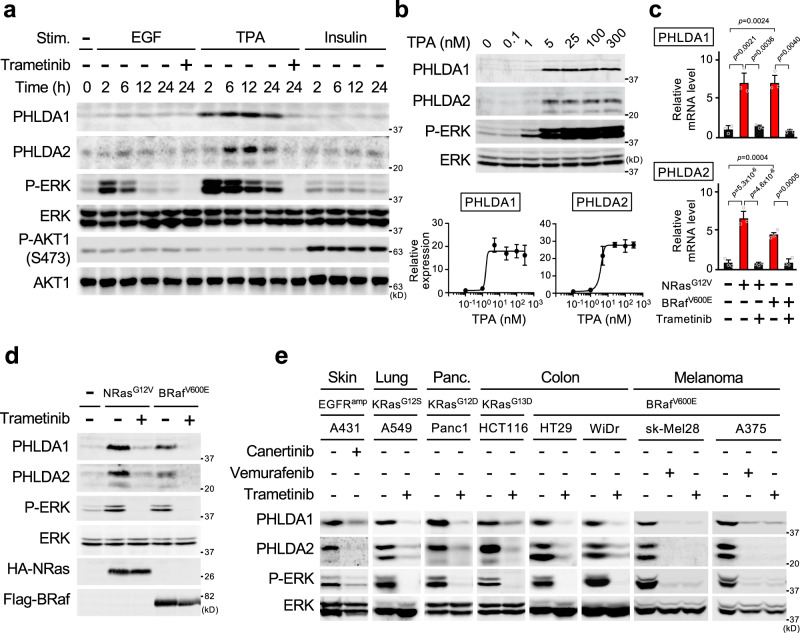
PHLDA1/2 are highly expressed in cancers harboring ERK activating oncogenes. **a** Oncogenic ERK signaling induces simultaneous PHLDA1/2 expression. HEK293 cells were stimulated with EGF, TPA, or insulin with or without trametinib for the indicated times. The cell lysates were probed for PHLDA1/2 expression, and for the phosphorylation states and protein levels of ERK and AKT by immunoblotting. **b** HEK293 cells were treated with various concentration of TPA for 6 h, and the cell lysates were analyzed as in (**a**). The intensity of the PHLDA1/2 bands was quantified (bottom graphs). **c**, **d** PHLDA1/2 mRNA and protein expression levels in HEK293 cells stably expressing NRas^G12V^ or BRaf^V600E^ were assessed using qRT-PCR (**c**) and by immunoblotting (**d**), respectively. Where indicated, the cells were pretreated with the MEK inhibitor, trametinib. **e** Immunoblot analysis of PHLDA1/2 expression in various cancer cell lines harboring the indicated oncogenes. Where indicated, cells were pretreated with the EGFR inhibitor (canertinib), the BRaf inhibitor (vemurafenib), or trametinib. **b**, **c** Data are mean ± SEM from three independent experiments. *P*-values were assessed using one-way ANOVA followed by Tukey’s multiple comparisons test. Source data are provided as a Source Data file.

The above gene-profiling experiments identified two out of the three AKT-inhibitor molecule PHLDA family members, PHLDA1 and 2, within the sigmoidal-B group of genes (Fig. 6c and Supplementary Fig. 8a). Although altered PHLDA1 expression in some clinical tumors has recently been reported^29^, the relationship between ERK signaling and PHLDA1/2 expression, and the role (if any) of the combined expression of PHLDA1/2 in cancer remain unknown. We therefore further analyzed these two genes. Initially, to test if the expression of PHLDA1/2 required hyperactivated ERK signaling, we treated HEK293 cells with the tumor promoter TPA or with EGF, which elicits strong and prolonged, or moderate and transient ERK activity, respectively, or with insulin, which preferentially induces AKT activity (Fig. 7a). TPA, but not EGF or insulin, efficiently induced PHLDA1/2 protein expression. We found that PHLDA1/2 induction exhibited a switch-like behavior in response to a gradual increase in ERK activity (Fig. 7b). Thus, these findings indicated that both the magnitude and the duration of ERK activity are critical determinants of PHLDA1/2 expression, and that their expression requires oncogenic (strong and constitutive) ERK activity.

**Fig. 8:**
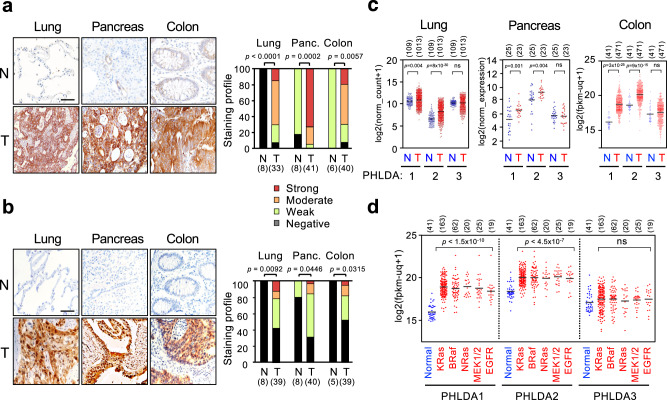
PHLDA1/2 proteins are highly expressed in human clinical cancers. **a**, **b** Representative immunohistochemical staining for PHLDA1 (**a**) and PHLDA2 (**b**) in tumor and corresponding normal tissues of lung, pancreas, and colon. Right graphs show the staining profiles of PHLDA1/2 in normal (N) and tumor (T) specimens. Scale bars, 60 μm. *P*-values were assessed using the Mann–Whitney *U* test. **c**, **d** Analyses of PHLDA1-3 mRNA expression in normal vs. various cancer tissues (**c**), and in normal vs. colon cancer tissues harboring mutations in the indicated oncogenes (**d**) based on the TCGA and GEO databases. Each horizontal bar represents the mean. Significance was determined using two-tailed Student t-test (**c**) or using one-way ANOVA followed by Tukey’s multiple comparisons test (**d**). ns, not significant. Numbers in parentheses indicate sample sizes. Source data are provided as a Source Data file.

Since various oncogenes lead to hyperactivation of ERK signaling, we next tested if oncogenes other than MEK1(K57N) also upregulate PHLDA1/2 expression. Introduction of NRas^G12V^ or BRaf^V600E^ into HEK293 cells evoked hyperactivation of ERK and upregulated PHLDA1/2 mRNAs (Fig. 7c) and proteins (Fig. 7d). Furthermore, human cancer cells harboring various oncogenes strongly expressed both PHLDA1/2 proteins. Inhibition of abnormal ERK activities in these cancer cells by canertinib (an EGFR inhibitor), vemurafenib (a BRaf inhibitor), or trametinib (a MEK inhibitor) treatment abrogated their expression (Fig. 7e). Thus, PHLDA1/2 are induced by various ERK-activating oncogenes and might therefore be overexpressed in various human cancers. Indeed, immunohistochemical analyses of clinical tumor samples demonstrated that PHLDA1/2 proteins were highly expressed in lung, pancreas, and colon cancers (Fig. 8a, b and Supplementary Fig. 8b, c). Analyses of large-scale cancer genomics datasets showed significantly higher expression of PHLDA1/2 mRNAs in various cancers than in their corresponding normal tissues (Fig. 8c). Furthermore, PHLDA1/2 were upregulated in association with the presence of ERK-activating oncogenes in colon cancers (Fig. 8d). Thus, PHLDA1/2 can serve as potential biomarkers for human cancers with ERK-activating oncogenes and may play roles in the pathophysiology of human cancers.

### PHLDA1/2 mediate cancer-specific ERK-AKT crosstalk and impact on the drug-sensitivity of tumor cells

**Fig. 9:**
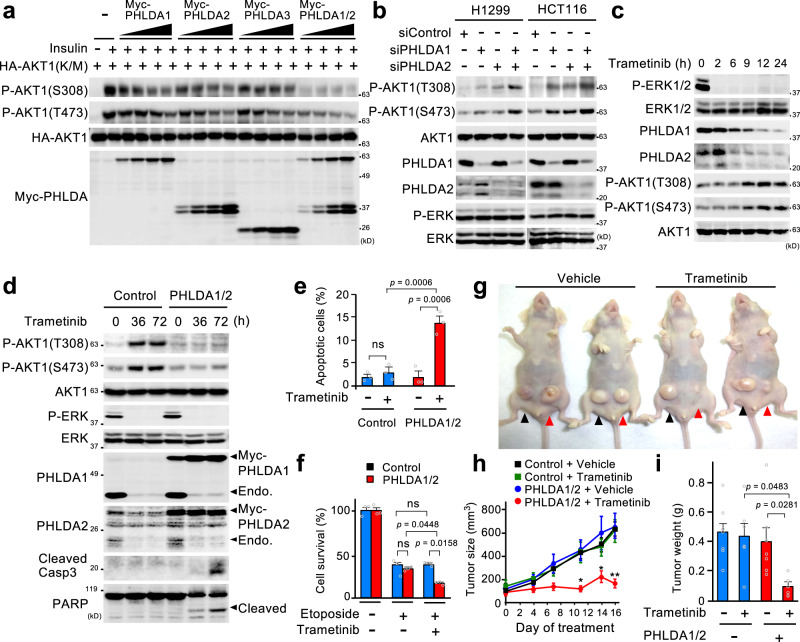
PHLDA1/2 mediate cancer-specific ERK-AKT crosstalk and impact on the drug-sensitivity of tumor cells. **a** Simultaneous expression of PHLDA1/2 is critical for efficient inhibition of AKT. HEK293 cells were transfected with inactive HA-AKT1(K/M), together with Myc-PHLDA1 and/or 2, or 3 as indicated. The cells were treated with insulin for 10 min, and the cell lysates were probed for AKT1 phosphorylated at T308 and S473 and for protein levels. **b** H1299 and HCT116 cells were transfected with siRNAs targeting PHLDA1 and/or PHLDA2. Expression levels of PHLDA1/2, the phosphorylation states of AKT and ERK, and their protein levels were analyzed by immunoblotting. **c**, **d** Trametinib-mediated PHLDA1/2 downregulation activates AKT. H1299 cells (**c**), and those stably expressing Myc-PHLDA1/2 or empty vector (control) (**d**) were treated with trametinib for the indicated times. Immunoblotting was performed as in **b**. In (**d**), apoptosis was monitored by immunoblotting of cleaved-caspase 3 and PARP. **e** The indicated cells were treated with trametinib (10 μM) for 72 h, and apoptosis was detected by annexin V staining. **f** The indicated cells were cultured with etoposide (1 μM) with or without trametinib (10 μM) for 4 days. The cell survival rate was determined by CCK8 assay. **e**, **f** Data are mean ± SEM (*n* = 3). *P*-values were assessed using one-way ANOVA followed by Tukey’s multiple comparisons test. ns, not significant. **g**–**i** H1299 control cells (black arrowheads) and H1299-PHLDA1/2 cells (red arrowheads) were transplanted into the left and right flanks of mice, respectively. Mice were then treated with vehicle or trametinib (3 mg/kg, 3 times a week) by oral gavage. **g** Representative images of the mice on day 16. **h** The growth curve of the xenograft tumors. **i** The weight of excised xenograft tumors on day 16 (vehicle, *n* = 7; trametinib, *n* = 6). Data are mean ± SEM. *P*-values were assessed using two-tailed Student t-test (**h**) or using one-way ANOVA followed by Tukey’s multiple comparisons test (**i**). In (**h**), *p < 0.05; **p < 0.01. Source data are provided as a Source Data file.

The PHLDA family proteins bind to various forms of phosphatidylinositols (PIPs) through a conserved PH domain, thereby compromising AKT activity, although their binding specificities for individual PIPs differ^30,31^. To investigate PHLDA1/2 roles in cancer, we first quantitatively compared the capacities of individual PHLDA proteins to suppress AKT activity. HEK293 cells were transfected with increasing amounts of Myc-tagged PHLDA1, 2, or/and 3, together with a constant amount of an HA-tagged kinase-defective AKT1(K/M) mutant. The transfected cells were stimulated with insulin to activate AKT and the activating phosphorylation of HA-AKT1(K/M) at S308 and T473 was monitored by immunoblotting (Fig. 9a). Although individual PHLDA isoform expression only weakly inhibited AKT activity, simultaneous PHLDA1/2 expression strongly inhibited it. Conversely, single depletion of endogenous PHLDA1 or PHLDA2 by their specific siRNAs only slightly enhanced AKT activity in cancer cells (Fig. 9b), whereas their co-depletion resulted in a much greater increase in AKT activity. Collectively, these findings indicate that co-expression of PHLDA1/2 exerts a synergistic effect on the inhibition of AKT activity, and that oncogene-induced hyperactivation of ERK signaling represses AKT activation in human cancer mainly through the simultaneous induction of PHLDA1/2.

The above findings imply that, when hyper-ERK activity in cancer is suppressed by ERK pathway-targeted therapeutics, PHLDA1/2 are downregulated and, therefore, AKT is activated by releasing from the PHLDA1/2-mediated inhibition. Indeed, treatment of H1299 cells with the MEK inhibitor, trametinib, rapidly abrogated ERK activity, thereby gradually decreasing the expression levels of PHLDA1/2 proteins (Fig. 9c). Importantly, AKT activity was incrementally increased in inverse proportion to the PHLDA1/2 downregulation. Virtually identical results were obtained with many other cancer cells (Supplementary Fig. 9a, b). To further confirm the functional relevance between the drug-induced PHLDA1/2 downregulation and AKT activation, we retrovirally introduced exogenous Myc-PHLDA1/2 into H1299 cells (termed H1299-PHLDA1/2 cells) to prevent their downregulation. As shown in Fig. 9d, stable expression of Myc-PHLDA1/2 completely abrogated the trametinib-induced AKT activation. Thus, treatment of cancer cells with ERK pathway-inhibitors enhances AKT activity mainly through PHLDA1/2 downregulation.

Since AKT signaling promotes cell survival, the ERK pathway-targeted drug-induced PHLDA1/2 downregulation and consequent AKT activation may prevent apoptosis of cancer cells and confer resistance to such anti-cancer agents. Indeed, in control H1299 cells, trametinib treatment failed to induce apoptosis, as measured by caspase-3 activation, PARP cleavage (Fig. 9d), and an annexin-V staining assay (Fig. 9e). Importantly, however, trametinib efficiently induced apoptosis, when the AKT activation was prevented by stable expression of PHLDA1/2 in H1299-PHLDA1/2 cells (Fig. 9d, e). Moreover, combination treatment of the genotoxic drug, etoposide, with trametinib did not further increase etoposide cytotoxicity towards H1299 cells compared with etoposide treatment alone, but it did do so towards H1299-PHLDA1/2 cells (Fig. 9f). Thus, ERK pathway-targeted drug-induced PHLDA1/2 downregulation profoundly attenuated not only the efficacy of ERK pathway-targeted therapeutics, but also the additive anti-tumor effect of conventional chemotherapies (e.g., etoposide) when combined with such drugs (e.g., trametinib) by enhancing pro-survival AKT activity in the tumor cells.

Next, to further validate these findings in vivo, we used a tumor xenograft treatment model. Control H1299 cells and H1299-PHLDA1/2 cells were subcutaneously injected into the left and right flanks of athymic nude mice, respectively. When tumors reached an average of 80 mm^3^ in volume, the mice were treated by oral gavage with vehicle or trametinib. As shown in Fig. 9g, h, and Supplementary Fig. 9c, these two cell lines exhibited similarly high levels of tumorigenicity in vehicle-treated mice. However, trametinib administration led to a drastic regression in the volume of tumors derived from H1299-PHLDA1/2 cells but not in that from control cells. Corresponding changes in tumor weight were observed after sacrifice of the mice (Fig. 9i and Supplementary Fig. 9d). These combined findings indicate that pharmacological inhibition of oncogenic ERK signaling in cancer induces the transactivation of AKT through PHLDA1/2 downregulation, thereby leading to resistance of cancer cells to ERK pathway inhibitors. These data further suggest that maintenance of PHLDA1/2 expression, if possible, would greatly enhances the anti-tumor efficacy of these drugs.

### Bortezomib potentiates the efficacy of ERK-targeted therapies by upregulating PHLDA1/2 expression

**Fig. 10:**
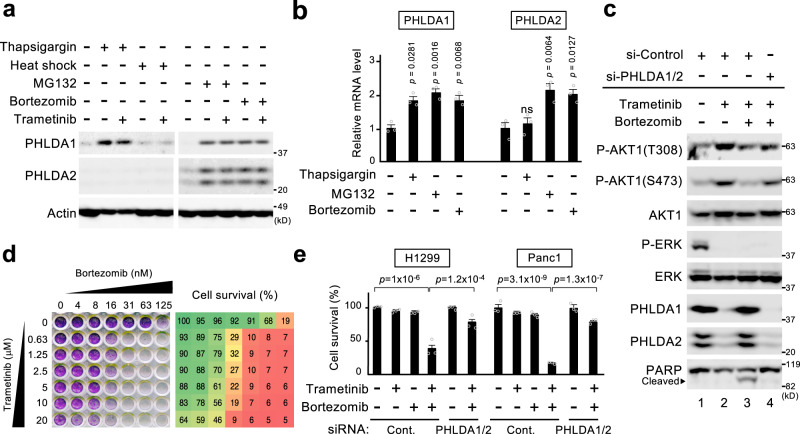
Bortezomib potentiates the efficacy of ERK-targeted therapies by upregulating PHLDA1/2 expression. **a**, **b** HEK293 cells were treated with thapsigargin (6 h), heat-shock (1 h), MG132 (6 h), or bortezomib (6 h) in the presence or absence of trametinib as indicated. PHLDA1/2 protein and mRNA expression levels were analyzed by immunoblotting (**a**) and qRT-PCR (**b**), respectively. **c** Retention of PHLDA1/2 expression by bortezomib prevents trametinib-induced AKT activation. H1299 cells that were transfected with siRNAs targeting PHLDA1/2 or control siRNA were treated with trametinib alone or in combination with bortezomib for 9 h. Expression levels of PHLDA1/2, the phosphorylation states of AKT and ERK, and cleaved PARP were monitored by immunoblotting. **d**, **e** MEK and proteasome inhibitors synergistically inhibit cancer cell growth. **d** H1299 cells were treated with various concentrations of bortezomib or trametinib alone or in combination for 72 h. The cells were then stained with crystal violet (left), and their survival rates were analyzed by CCK8 assay (right). **e** H1299 or Panc1 cells were transfected with siRNAs targeting PHLDA1/2 or with control siRNA, were treated with trametinib (10 μM) and/or bortezomib (10 nM) for 72 h, and their survival rates were then analyzed as in **d**. **b**, **e** Data are mean ± SEM from three independent experiments. *P*-values were assessed using one-way ANOVA followed by Tukey’s multiple comparisons test. Source data are provided as a Source Data file.

The above findings prompted us to develop a therapeutic strategy to maintain PHLDA1/2 expression even in the presence of ERK pathway inhibitors. In this respect, heat shock or an endoplasmic reticulum (ER) stressor, thapsigargin, were shown to upregulate at least PHLDA1 in certain cell types^32,33^. We therefore first investigated if these or other stressors could induce PHLDA1 and/or PHLDA2 expression in HEK293 cells. Heat shock induced neither of the PHLDAs, and thapsigargin upregulated only PHLDA1, but not PHLDA2 (Fig. 10a, b). However, the proteasome inhibitors MG132 and bortezomib, which also provoke ER stress, significantly upregulated PHLDA1 and PHLDA2 protein and mRNA expression in HEK293 and in various cancer cells (Fig. 10a, b and Supplementary Fig. 9e). These proteasome inhibitors augmented PHLDA1/2 expression even in the presence of trametinib, indicating ERK-independent induction (Fig. 10a). Furthermore, these drugs upregulated PHLDA1/2 not only transcriptionally but also post-transcriptionally, as they suppressed the degradation of PHLDA1/2 proteins and thus greatly extended their half-lives (Supplementary Fig. 9f).

We next examined the effect of bortezomib on the ERK-pathway targeted drug-induced transactivation of AKT in cancer cells. Treatment of H1299 cells with trametinib alone elevated AKT activation by downregulating PHLDA1/2 (Fig. 10c, lane 2). In contrast, following co-administration of bortezomib with trametinib, PHLDA1/2 expression was not downregulated even under conditions of low ERK activity, and the unwanted AKT activation was thereby prevented, resulting in efficient induction of apoptosis, as measured by PARP cleavage (Fig. 10c, lane 3 and Supplementary Fig. 9g). We confirmed that bortezomib repressed AKT activity via PHLDA1/2 upregulation, as depletion of PHLDA1/2 by siRNAs restored the AKT activation even in the presence of bortezomib (Fig. 10c, lane 4). Consistent with these biochemical data, treatment with either trametinib or bortezomib alone showed only minor inhibitory effects on H1299 cell growth, whereas their combined treatment exhibited a synergistic, more potent cytotoxic effect (Fig. 10d, e). Again, the enhanced anti-tumor efficacy of this combination therapy was abrogated by siRNA-mediated PHLDA1/2 depletion and the consequent AKT activation (Fig. 10e). Essentially identical results were also observed in the pancreatic cancer cells, Panc1 (Fig. 10e). Thus, co-administration of bortezomib with ERK pathway inhibitors (e.g., trametinib) can maintain an elevated level of PHLDA1/2 expression in cancer cells, and greatly enhance the anti-tumor effects of ERK pathway-targeted drugs by disrupting the PHLDA1/2-mediated, cancer-specific aberrant signaling crosstalk between ERK and AKT (Supplementary Fig. 9h).

## Discussion

In this study, we present the crystal structure of a Cmut at 2.0 Å resolution and demonstrated the different biological and biochemical properties of the disease-associated MEK1 mutants. In general, Rmuts are moderately active and are thus non-oncogenic, whereas Cmuts possess strong and oncogenic kinase activity. The properties of these two classes of MEK1 mutants are due to their different activation mechanisms. Although all the disease-associated MEK1 mutants exhibited moderate kinase activity independent of MEK1 T-loop phosphorylation as confirmed by the higher kinase activities of their T-loop unphosphorylatable AA mutants versus WT MEK1, the cancer-, but not the RASopathy-, associated mutants can additionally autophosphorylate their own T-loops, thereby leading to strong and oncogenic kinase activities. On the basis of our structural analysis of MEK1(C121S), we propose the following model for the molecular basis of the constitutive but different activities of the MEK mutants. Unlike previous assumptions based on in silico modeling^17,34^, destabilization of the helix A by the MEK1 mutation leads to an excessive outward, but not inward, rotation of the helix C (i.e., the αC-extra-out conformation). This outward displacement allows the helix C to create a coiled-coil-mediated stable interaction with the α helix in the T-loop, thereby converting the T-loop from a closed to an open conformation. Since the resulting extended conformation of the T-loop uncovers the MEK1 substrate binding region and the active-site cleft, both of which are vital for catalysis, it is most likely a primary cause of the phosphorylation-independent activation of disease-associated MEK1 mutants. Furthermore, our structural and biochemical analyses revealed that the disruption of the interdomain H-bonds connecting H119 and F129 with K57 is critical for the abnormal autophosphorylating activity of the Cmuts. Thus, only mutations that lead to severe distortion of the local structure around the H-bonds can elicit this autophosphorylation. Our findings explain why only specific types of mutations enable MEK1 to undergo autophosphorylation and induce carcinogenesis.

Another important finding of our structural analysis is that C121S mutation deforms the allosteric, but not the ATP-binding pocket of MEK1. Since all the MEK1 mutants tested exhibited resistance to its allosteric inhibitors, other disease-associated MEK1 mutations may also lead to similar structural distortion in their allosteric pockets, thereby acquiring insensitivity to these inhibitors. C121S mutation, however, scarcely altered the ATP-binding pocket structure. In this regard, we found that the ATP-competitive MEK inhibitor, hymenialdisine, more potently suppressed the abnormal kinase activity of MEK1(C121S) than that of WT (Supplementary Fig. 4h), and efficiently inhibited the growth of A375 cells expressing any of the MEK1 mutants tested (Supplementary Fig. 4g). Thus, the development of more effective ATP-competitive inhibitors for MEK, or of new allosteric inhibitors that fit the deformed allosteric pocket, is of paramount importance for the treatment of human cancers with MEK mutations such as lung and colon cancers, and melanomas.

We further showed here that the two classes of MEK1 mutations differentially altered the spatiotemporal properties of ERK signaling: The Rmuts increased the magnitude and duration of growth factor-induced ERK activity and led to the prolonged nuclear accumulation of ERK, whereas the Cmuts elicited persistent activation and nuclear translocation of ERK irrespective of stimulation. Interestingly, these two different alterations in ERK signaling dynamics generated distinct expression profiles of specific sets of genes in cells that are related to the pathophysiology of either RASopathies or cancers. Thus, the RASopathy- and cancer-associated mutations do not simply lead to quantitative differences in the basal kinase activity of MEK1, but they induce qualitative differences in the spatiotemporal dynamics of ERK signaling and in consequent gene expression profiles, thereby leading to the development of distinct clinical manifestations (Supplementary Fig. 6e).

Our transcriptome analyses of the F53S and K57N cells showed that genes affected by the mutants exhibited one of at least five distinct expression patterns relative to the degree of aberrant ERK activity. Of these patterns, the genes in the bell-shaped and sigmoidal-B groups are specifically expressed in F53S and K57N cells, respectively, and therefore might be useful as diagnostic markers and/or therapeutic targets for RASopathies and cancers, respectively. Indeed, the sigmoidal-B group of genes included various cancer-specific molecules known to be tumor-associated or cancer-testis antigens. Although the transcriptional regulation of these tumor antigens has not been well-studied, our data indicate that hyper-ERK activity in cancer contributes to their abnormal expression. Since some of these cancer- and RASopathy-signature genes encode secreted or transmembrane proteins, these molecules may serve as serum (or amniotic fluid) diagnostic markers specific for cancer and RASopathies, and as targets for the development of antibody-based or chimeric antigen receptor (CAR) T cell therapies for cancer. Furthermore, the TFBS analysis of K57N-UGs showed that, among ERK substrate TFs, oncogenic ERK signaling preferentially activated the SP and JUN family members and was less effective in ELK1 or Myc activation. In this respect, a previous study demonstrated that, following mitogenic stimulation, although ERK-mediated rapid phosphorylation of ELK1 at T369 and S384 enhances its transcriptional activity, slow ELK1 phosphorylation at T337, T418, and S423 by ERK does not activate but rather inhibits its transcriptional response^35^. Therefore, constitutive ERK activation likely induces the inhibitory, slow ELK1 phosphorylation and attenuates its transcriptional activity, possibly explaining why oncogenic ERK signaling less efficiently upregulates ELK1 target genes. Likewise, inhibitory phosphorylation of Myc has also been reported^36^. Thus, oncogenic ERK signaling drives an altered transcriptional program by preferentially activating specific ERK-regulated TFs to generate the cancer-specific gene expression pattern.

We also showed here that PHLDA1/2 are simultaneously induced by oncogenic, but not by physiological ERK signaling, and that both proteins are indeed upregulated in various human clinical cancers. Importantly, while single expression of either PHLDA1 or 2 exhibited only a marginal effect on AKT activity, their combined expression profoundly repressed it in cancer cells. This synergistic inhibitory effect of PHLDA1/2 may be explained by their different binding specificities for PIPs: PHLDA1 predominantly binds to PIP3 and PI(3,5)P2, but not to PI(3,4)P2^30^, while PHLDA2 can interact more widely with various PIPs, including PI(3,4)P2, PIP3, and PI monophosphates^31^. Since PI(3,4)P2 and PIP3 bind to and activate AKT^37^, the combined expression of PHLDA1/2 would more strongly inhibit AKT activation by simultaneously sequestering these and other PIPs. Even more importantly, ERK-induced PHLDA1/2 expression serves as a double-edged sword for cancer prevention and treatment. ERK-induced PHLDA1/2 upregulation in pre-treated cancer cells inhibits AKT, which may somewhat suppress tumor cell survival and progression in patients. However, when the patients are treated with ERK pathway-targeted therapeutics, PHLDA1/2 levels are downregulated in cancer cells, leading to the unwanted enhancement of AKT activity and associated cell survival, and thereby to resistance to the anti-cancer agents (Supplementary Fig. 9h). In order to overcome these adverse effects, we identified bortezomib as a drug that can upregulate PHLDA1/2 expression independently of ERK activity. We found that the combination treatment of trametinib with bortezomib prevented trametinib-induced PHLDA1/2 downregulation and the subsequent AKT transactivation in cancer cells, thereby exerting a synergistic potent anti-cancer effect sufficient to induce apoptosis.

Recently, combination therapy with ERK pathway- and AKT pathway-inhibitors has emerged as a promising strategy for cancer treatment. Clinical trials of such therapies, however, have failed to yield encouraging results because of intolerable side effects caused by systemic inhibition of these two critical pathways^38^. Since PHLDA1/2, which inhibit AKT, are specifically upregulated in cancer cells, the retention of their increased expression is an ideal therapeutic target to enable cancer-specific AKT inhibition. Thus, the combination of ERK pathway inhibitors with drugs such as bortezomib that retain PHLDA1/2 expression in cancer would be a potential strategy to achieve efficient tumor regression with fewer side effects.

## Methods

### Plasmids

The pcDNA3 vector (Invitrogen) was used to generate expression plasmids for 3xHA- or 5xMyc-tagged MEK1, MEK2, ERK2, PHLDA1, PHLDA2, PHLDA3, AKT1 and their derivative mutants. The MEK1 mutants derived from RASopathy or sporadic cancers were generated using PCR-directed mutagenesis. pCold (Takara Bio), pRSF-Duet (Merck-Millipore) and pGEX-6P vectors (GE Healthcare) were used for bacterial expression of GST- or His-tagged MEK1, MEK2, BRaf, ERK2 and their mutant forms. Catalytically inactive rat ERK2(K/N) was generated by replacing Lys52 with an Asn codon. Catalytically inactive MEK1(K/M), MEK2(K/M), and AKT1(K/M) mutants were generated by replacing Lys97, Lys101, and Lys179, respectively, with a Met codon. The retroviral expression vectors, 3xHA-MEK1(WT or mutants), 5xMyc-PHLDA1, 5xMyc-PHLDA2, 3xHA-NRas^G12V^, or 3xFlag-BRaf^V600E^ were generated by subcloning into a pQCXIP or pQCXIH vector (Invitrogen). The ERK1-GFP construct was generated by fusing the human ERK1 ORF to the N-terminus of GFP, and the chimeric coding region was cloned into a pQCXIH vector. For utilization of the Shield1-dependent protein stabilization system, the DD sequence (destabilizing domain; Clontech) was fused to the N-terminus of active MEK1(S218D/S222D), and the fused construct was cloned into a pQCXIP vector. DNA constructs generated by the authors will be distributed upon request to other research investigators under a Material Transfer Agreement.

### Media and buffers

Lysis buffer A contained 20 mM Tris-HCl (pH 7.5), 137 mM NaCl, 1% Triton X-100, 0.5% deoxycholate (DOC), 10% glycerol, 2 mM EDTA, 50 mM β-glycerophosphate, 1 mM sodium vanadate, 10 mM NaF, 1 mM dithiothreitol (DTT), 1 mM phenylmethylsulphonyl fluoride, 10 μg/ml aprotinin and 10 μg/ml leupeptin. Lysis buffer B is the same as lysis buffer A except that DOC is omitted. Kinase buffer for the in vitro kinase assay contained 25 mM Tris-HCl (pH 7.5), 25 mM MgCl_2_, 10 mM β-glycerophosphate, 5 mM sodium vanadate, and 2 mM DTT.

### Mouse xenograft

Five-weeks-old female nude mice (BALB-c/nu) were purchased from Oriental Yeast (Tokyo, Japan). H1299 (control or PHLDA1/2) stable cell lines were suspended in DMEM (FBS free) and mixed with Matrigel (BD Biosciences) at a ratio of 1:1. The cell mixture containing 1 × 10^7^ cells was injected subcutaneously into the left or right side flank of each mouse. When the average size of tumors reached 80 mm^3^, the mice were separated into two groups. Trametinib was diluted in 0.5% HPMC (Sigma) with 0.2% Tween-80, and was applied by oral gavage 3 times a week (3 mg/kg, 6 mice). The same volume (200 μL) was orally administrated to the vehicle control group at the same dosing frequency (7 mice). Tumor volume (mm^3^) was measured with calipers and was calculated as (L x W^2^) / 2, where L is length and W is width. The animal experiments in this study were approved by the animal experiment committee at the Institute of Medical Science, The University of Tokyo (IMSUT) (approval number: A16-5), and animal care was conducted in accordance with institutional guidelines. The endpoints for mice were tumor weight exceeding 10% or more of body weight, interference with basic/vital bodily functions, or persistent ulceration. At no point did any mice exceed maximal tumor burden. The housing conditions for the mice are as follows: temperature 22 ± 2 °C, humidity 55 ± 5%, and light/dark cycle 12 h/12 h (8 a.m.−20 p.m. light).

### Cell culture and transfection

A375 (CRL-1619), Sk-Mel28 (HTB-72), H1299 (CRL-5803), and HT29 (HTB-38) were obtained from ATCC. HEK293 (RCB1637), G361 (RCB0991), A549 (RCB3677), T24 (RCB2536), A431 (RCB0202), Panc1 (RCB2095), HCT116 (RCB2979), and GP2-293 (RCB2354) were obtained from RIKEN cell bank. WiDr (JCRB0224) was obtained from Japanese Cancer Research Resources Bank (JCRB). Plat-E cells and *MEK1*^−/−^ MEFs were originally generated and provided by T. Kitamura (University of Tokyo) and by J. Charron (Université Laval, Québec), respectively. WiDr (ICLAC-00103) has been reported to be a derivative of HT29 and this fact is described in the Cell Model Passport (https://cellmodelpassports.sanger.ac.uk) and the JCRB Cell Bank database (https://cellbank.nibiohn.go.jp/~cellbank/en/search_res_det.cgi?ID = 308#), but it was included in our analysis for diversity owing to its oncogene status and tissue origin. Cells were maintained in Dulbecco’s modified Eagle medium (DMEM) supplemented with 10% fetal bovine serum (FBS), L-glutamine, penicillin, and streptomycin, except that HCT116 cells were cultured in McCoy’s 5a Medium with 10% FBS, L-glutamine, penicillin, and streptomycin. Cells were treated with EGF (5 ng/mL), TPA (100 nM), insulin (200 nM), thapsigargin (1 μM), heat-shock (43 °C), MG132 (10 μM), or bortezomib (10 μM) for 10 min or for the indicated times. Cells were incubated with 10 μM U0126, canertinib, vemurafenib or trametinib for 24 h unless otherwise noted. For transient transfection assays, HEK293 cells grown on 35 mm dishes in DMEM (10% FBS) were transfected with the appropriate combinations of expression plasmids using the X-tremeGENE 9 DNA transfection reagent (Sigma). The total amount of plasmid DNA was adjusted to 1 μg/dish with the empty vector DNA (pcDNA3). The cells were harvested 48 h after transfection. HEK293 stable cell lines expressing HA-MEK1, Flag-BRaf, HA-NRas, or their mutant derivatives were generated by transfection of their expression plasmids (pQCXIP) and then puromycin selection.

### Retroviral infection

Stable cell lines derived from H1299 cells were generated by retroviral infection. Retroviruses were produced in GP2-293 packaging cells by transient transfection with pVSV and either pQCXIP or pQCXIH plasmids. Culture supernatants were collected 48 h post-transfection, filtered, and supplemented with 8 μg/ml polybrene. Cells were infected with retrovirus and selected with puromycin or hygromycin. Retroviral infection of MEFs was performed as described above, except that Plat-E packaging cells were used.

### Protein preparation or structural analysis

The gene encoding the F11 fragment of human MEK1^20^ with codon optimization for expression in *E. coli* was chemically synthesized by Genewiz. The synthesized gene was amplified by PCR and cloned into the pET11a vector (Novagen). Mutations leading to the substitution of Cys121 with Ser were introduced using PCR-mediated site-directed mutagenesis. The constructs were sequenced to confirm their identities. *E. coli* strain BL21(DE3) cells were used for expression of recombinant proteins. For crystallization, MEK1 was first purified using a Ni-NTA column (QIAGEN). After affinity chromatography, the protein was purified on a HiLoad 26/60 Superdex 200 PG column (GE Healthcare) eluted with 20 mM Tris-HCl (pH 8.0) and 150 mM NaCl.

### X-ray crystallography

Crystallization trials were performed with the sitting-drop vapor-diffusion method at 20 °C. Drops (0.3 μl) of 10 mg/ml MEK1(C121S) in 20 mM Tris-HCl (pH 8.0), 150 mM NaCl, 10 mM DTT, and 2 mM AMPPNP were mixed with equal amounts of reservoir solution consisting of 26% polyethylene glycol 3350, 100 mM BICINE (pH 9.0), 150 mM NH_4_F, and 1% glycerol and were equilibrated against 40 μl of the same reservoir solution by vapor diffusion. Crystals were cryoprotected with a reservoir solution supplemented with 6% glycerol, flash-cooled, and were kept at −173 °C during data collection. Diffraction data were collected at a SPring-8 beamline BL32XU using a wavelength of 1.0000 Å (Supplementary Table 1). Data were processed using the program XDS (ver. May 1, 2016). The structure of MEK1(C121S) was determined by the molecular replacement method using the BALBES server, where the structure of human MEK1 (PDB ID 3EQC) was used as a search model. Further model building was performed manually using the program COOT (ver. 0.8.9), and crystallographic refinement was performed using the program Phenix (ver. 1.11.1). Ramachandran plot using Rampage shows 296, 4, and 1 residues are located in favored, allowed, and outlier regions, respectively. The statistics of X-ray crystallography are summarized in Supplementary Table 1. All structural models used in figures were prepared using the program PyMOL (ver. 2.1.0). The crystal structure of MEK1(C121S) has been deposited in the Protein Data Bank (PDB) under the accession codes 7F2X.

### Purification of recombinant proteins

The *E. coli* strain DH5α was transformed with a pGEX-6P-based plasmid encoding GST-ERK2(K/N) or the WT or mutant forms of GST-MEK1 and GST-MEK2. Exponentially growing DH5α cells carrying one of these plasmids were incubated with 0.5 mM IPTG at 25 °C for 14 h before harvesting. Cells were suspended in cold PBS, lysed by sonication, and then clarified by centrifugation (30,000 g for 15 min at 4 °C). The clear supernatant was filtered through a 0.45 μm filter, and GST-tagged proteins were purified using a GSTrap HP affinity column. pRSF-Duet encoding 6xHis-BRaf^V600E^ was transduced into BL21(DE3) cells and induced by addition of IPTG (1 mM final concentration) at 25 °C for 14 h. DH5α cells carrying a pCold-based plasmid encoding 6xHis-MEK1(K/M) or MEK2(K/M) were incubated with 0.5 mM IPTG at 15 °C for 24 h. After sonication and centrifugation, the cleared cell lysates were loaded onto a HisTrap HP column, and His-tagged proteins were affinity purified. The purified proteins were analyzed by Western blotting or were used for in vitro kinase assay.

### In vitro kinase assay

To assess the kinase activity of MEK1, purified MEK1 (0.6 μg) was mixed with inactive ERK2(K/N), MEK1(K/M), or MEK2(K/M) proteins (0.6 μg) in kinase buffer (40 μl) containing 160 μM ATP, and the mixtures were incubated at 26 °C for 7 min unless otherwise indicated. Phosphorylated MEK1 was obtained by incubating purified MEK1 (0.6 μg) with recombinant active Raf-1ΔN or BRaf^V600E^ proteins (0.6 μg) at 26 °C for 7 min. For the MEK1 immunoprecipitation kinase assay, transfected HEK293 cells were lysed with lysis buffer B and were incubated with anti-HA antibody (3F10) for 3 h at 4 °C. The precipitates were recovered with protein G-Sepharose beads and were washed three times with lysis buffer B and twice with kinase buffer. Immunoprecipitates were resuspended in 24 μl of kinase buffer containing GST-ERK2(K/N) (0.6 μg). The kinase reaction was initiated by the addition of ATP (final concentration, 160 μM). Following a 7 min incubation at 26 °C, the reactions were terminated by the addition of SDS loading buffer. Phosphorylated ERK or MEK in the reaction samples was analyzed by immunoblotting using their phospho-specific antibodies. The phosphorylation levels were quantified using the luminescent image analyzer, LAS-1000 Plus (Fujifilm).

### Immunoblotting analysis

Digitized images were captured by LAS-1000 Plus or LAS-4000 with a CCD (charge-coupled device) camera (Fujifilm). The following primary antibodies were used: Monoclonal anti-HA F-7 (Santa Cruz Biotechnology, sc-7392), anti-GST B-14 (sc-138), anti–Myc 9E10 (sc-40), anti-TFPI2 B-7 (sc-48380), anti-MEK1 H-8 (sc-6250), anti-HA 16B12 (Covance, MMS-101R), anti-HA 3F10 (Roche, 11867423001), anti-Flag M2 (Sigma–Aldrich, F1804), anti-phospho-Raf-1 56A6 (Cell Signaling Technology, 9427), anti-phospho-MEK1/2 41G9 (9154), anti-phospho-AKT1(T308) 244F9 (4056), anti-phospho-AKT1(S473) D9E (4060), anti-phospho-S6 D57.2.2E (4858), anti-S6 5G10 (2217), anti-MEK2 13E3 (9147), anti-GDF15 D2A3 (8479), anti-PHLDA1 EPR6674 (Abcam, ab133654), anti-PHLDA2 (ab58379), Anti-β-Actin 6D1 (FUJIFILM Wako, 010-27841); polyclonal anti-ERK (Santa Cruz Biotechnology, sc-94), anti-C-Raf-1 (sc-227), anti-B-Raf (sc-166), anti-phospho-ERK1/2 (Cell Signaling Technology, 9101), anti-AKT (9272), anti-cleaved caspase3 (9661), anti-PARP (9542), anti-COL14A1 (Abcam, ab101464), anti-TM4SF1 (ab113504), anti-PHLDA2 (Proteintech, 14661-1-AP), anti-TM4SF19 (Sigma-Aldrich, SAB1102826), anti-EMP1 (SAB1302714). For GDF15, TFPI2 and COL14A1 immunoblots, cells were incubated for 72 h in serum-free DMEM, cell culture supernatants were then concentrated using an Amicon Ultra-15 (Merck Millipore), and 2.4 μg of total protein per lane was electrophoresed under reducing conditions. For the detection of transmembrane proteins (TM4SF1, TM4SF19 and EMP1) by immunoblotting, deglycosylation was performed by incubating cell lysates with Glycopeptidase F (Takara Bio) at 37 °C overnight. All primary antibodies were used at a dilution of 1:1,000. The following second antibodies were used: Anti-mouse HRP antibody (NA931, Cytiva; at a dilution of 1:5000); anti-rabbit HRP antibody (NA934, Cytiva; at a dilution of 1:2500).

### Immunofluorescence microscopy

HEK293 cells growing on glass coverslips were transiently transfected with an expression plasmid encoding HA-MEK1 or its mutant derivatives. Eighteen hours after transfection, the cells were fixed with 3% paraformaldehyde in PBS (pH 7.4) for 10 min. After washing with PBS, the cells were permeabilized with 0.1% Triton X-100 for 5 min, washed again, and then incubated in BlockAce (Yukijirushi) at RT for 1 h. The cells were incubated with 1 μg/ml of the anti-HA antibody 16B12 (MMS-101P, Covance) at RT for 50 min in PBS containing 2% BSA. The cells were washed four times with PBS and were then incubated with Alexa-Fluor 488 goat anti-mouse IgG_1_ (A-21121, Molecular Probe; 1:2000) at RT for an additional 30 min. The coverslips were washed four times with PBS and mounted in FluorSave (Calbiochem). Image J software (ver. 1.53a) was used for image analysis.

### Cell proliferation assays

For the proliferation assay, 1000 cells/well were seeded in triplicate in DMEM with 10% FBS, and cell growth was monitored each day using the Cell Counting Kit-8 (Dojindo). For the anchorage-independent growth assay in soft agar, cells were cultured in 60 mm dishes previously covered with soft agar (SeaPlaque Agarose) as the bottom layer (DMEM with 0.5% agar and 10% FBS). The top layer contained MEFs (30,000 cells) with 0.35% agar and 10% FBS. Medium was added to the top layer to prevent drying of the agarose gel. MEFs were cultured for 3 weeks, and colonies (>0.1 mm) were counted. The mean of colony numbers was calculated from triplicate assays.

### Growth inhibition assays

Cells were seeded onto 96-well plates at a density of 3000 cells per well. Following adherence of the cells, DMSO or drugs were added into each well, and the cells were incubated for 3 (H1299 and Panc1) or 4 (A375) days. Cell viability was measured using the Cell Counting Kit-8 (Dojindo, CCK-8 assay) and was calculated as a percentage of the control (DMSO) after background subtraction. The experiments were carried out in triplicate. For the analysis of drug-resistance mutations of MEK1, data were fitted to sigmoidal dose–response curves, and the half maximal growth inhibitory concentration (GI_50_) value was calculated using GraphPad Prism 5.0 (GraphPad software).

### Immunohistochemistry

Three kinds of cancer tissue microarrays (pancreas, PA484; lung, LC483; and colon, CO483) were purchased from US Biomax. The tissue slides were deparaffinized and subjected to antigen retrieval by incubating in citrate buffer (pH 6) at 95 °C for 40 min. Endogenous peroxidase activity in each tissue sample was blocked by treatment with 0.3% hydrogen peroxide. The rehydrated slides were incubated with BlockAce (Yukijirushi) for 10 min at RT, and incubated with an anti-PHLDA1 (Abcam, ab133654; 1:50) or an anti-PHLDA2 antibody (Abcam, ab58379; 1:50) at 4 °C overnight. Anti-rabbit or -mouse HRP antibody (Dako Envision systems) and a chromogen DAB (3,3’-Diaminobenzidine) was used for visualizing the interaction between the first antibody and antigen. The total PHLDA1 and PHLDA2 immunostaining scores were calculated as the product of (1) the staining intensity and (2) the percentage of positive cells. (1) Intensity was quantified as follows: 0, negative (no staining), 1, weak staining (detection required a high magnification); 2, moderate staining (readily detected at a medium magnification); 3, strong staining (readily detected at a low magnification). (2) The percentages of positive cells were scored into four categories: 0, 0%; 1, 1–33%; 2, 34–66%; 3, 67–100%. The final score for the staining of PHLDA1 or PHLDA2 was defined as negative (final staining score = 0), weak expression (final staining score = 1 or 2), moderate expression (final staining score = 3 or 4), and strong expression (final staining score ≥ 6). Differential expression between normal and malignant tissues was evaluated using the Mann–Whitney *U* test. Immunohistochemical evaluation was performed by a pathologist (D.M.) using light microscopic observations.

### Detection of apoptotic cells

Apoptotic cells were detected using the Annexin V-Cy3 Apoptosis Kit (Biovision) according to the manufacturer’s instructions. Briefly, cells were collected, resuspended in 1x binding buffer, and then incubated with annexin V-Cy3 in the dark for 5 min. Annexin V-Cy3-bound cells stained in the plasma membrane were detected by fluorescence microscopy and counted. The experiments were carried out in triplicate.

### DNA microarray and pathway analyses

Total RNA was purified from each HEK293 cell line using the miRNeasy kit (QIAGEN), and was then reverse-transcribed using the PrimeScript RT Master Mix (Takara Bio) according to the manufacturer’s instructions. After confirming the purity of RNA samples, microarray analysis was performed using the Agilent SurePrint G3 Human GE microarray 8x60K v2 kit (Agilent). The averaged expression level of each gene was calculated from three independent experiments. Microarray data in this study were deposited in the Gene Expression Omnibus (GEO) database under accession number GSE165823.

To find pathways responsible for observed differential gene expression, upregulated or downregulated genes with a fold change of >1.2 in MEK1(F53S)-expressing cells or of >2.5 in MEK1(K57N)-expressing cells relative to MEK1(WT)-expressing cells were subjected to functional annotation analysis using the Database for Annotation, Visualization and Integration Discovery (DAVID) database (http://david.abcc.ncifcrf.gov). The negative logarithm of each *p*-value for the significant KEGG pathways (*p* < 0.1) is shown.

### Gene expression analysis using public data

To compare the expression level of PHLDA family genes between normal and tumor tissues, TCGA clinical and gene expression data of lung (TCGA-LUNG) and colon (GDC-TCGA-COAD) were retrieved from the UCSC cancer genome browser (https://genome-cancer.ucsc.edu). The expression data of PHLDA family genes in pancreas (GSE1542) was obtained from the publicly available NCBI GEO database. In Supplementary Fig. 7a–c, the expression data of the sigmoidal-B group of genes was based on the TCGA-TARGET-GTEx dataset.

### GSEA and TransFac analyses

GSEA was used to interpret gene expression data by determining the statistical significance of differences in predefined gene sets between biological states. All expression data of mRNAs derived from the cells expressing MEK1(F53S) or MEK1(K57N) were used to generate the expression dataset. GSEA was performed on the statistically significant gene list of the c1, c2, c3, c4, c5, c6, and c7 gene sets from the Molecular Signatures Database (MSigDB) using GSEA software V3.0 (Broad Institute, MIT, Cambridge, MA, USA). TransFac analysis was performed using the genes that were upregulated by >2-fold in the HEK293 cells expressing MEK1(K57N) compared to cells expressing WT MEK1. The region from −10,000 bp upstream to +1000 bp downstream of the transcription start site of each gene was analyzed. A *p*-value of <0.05 was considered statistically significant.

### RNA extraction and qRT-PCR analysis

Total RNA was extracted using a standard TRIzol extraction protocol, and cDNAs were obtained by reverse transcription using the PrimeScript RT Master Mix (Perfect Real Time) (Takara Bio). Real-time quantitative PCR (qRT-PCR) analysis was performed using the Thermal Cycler Dice real time system (Takara Bio) and the Thunderbird SYBR qPCR mix (Toyobo). The relative gene expression levels were calculated by normalizing to that of *GAPDH*. All qRT-PCR experiments were performed in triplicate, and the mean ± SEM is shown. The sequences of primers used for qRT-PCR are listed in Supplementary Table 2. Primer design and specificity check were performed using primer-BLAST (https://www.ncbi.nlm.nih.gov/tools/primer-blast/). To establish the models of gene-expression patterns regulated by ERK signaling, we calculated gene expression levels in the cells with MEK1(F53S or K57N) as fold change (FC) in relation to those in MEK1(WT), and evaluated these FC values based on the following criteria: Linear increase; 1 < FC < 2.5, sigmoidal increase; FC ≥ 2.5, and decrease; FC < 1.

### siRNA transfection experiments

H1299 and HCT116 cells were plated on a 6-well plate at 30% confluence and were transfected with MISSION siRNA Universal Negative Control (Sigma) or siRNAs targeting PHLDA1 (siRNA ID: SASI_Hs01_00161315, Sigma) or PHLDA2 (siRNA ID: SASI_Hs01_00085404, Sigma) using Lipofectamine RNAiMAX (Invitrogen). Three days later, the cells were lysed and the expression of PHLDA1/2 was checked by immunoblotting.

### Statistics and reproducibility

The statistical significance of the difference between mean values was tested using two-tailed Student’s t-test or one-way ANOVA with Tukey’s multiple comparisons test (***p < 0.001; **p < 0.01; *p < 0.05) as described in the figure legends. For comparative analysis of immunostaining between normal and malignant tissues (Fig. 8a, b), differential expression was evaluated using the Mann–Whitney *U* test. Data are presented as means ± SEM. For in vitro and in vivo analyses, all the experiments were repeated independently at least three times with similar results (Figs. 1b, c, f–l; 3b; 4a; 5g, 6e, f; 7a, b, d, e; 9a–d; 10a, c; Supplementary Figs. 1c, f, g, h–o; 2b, d, f; 3e; 4a–e, f, h; 5a, c–e; 6b, d; 7d–f; 9a, b, e–g).

### Reporting summary

Further information on research design is available in the Nature Research Reporting Summary linked to this article.

## Supplementary information

Supplementary Information

Description of Additional Supplementary Files

Supplementary Movie 1

Supplementary Movie 2

Supplementary Movie 3

Reporting Summary

## Data Availability

The microarray data generated in this study has been deposited in the Gene Expression Omnibus (GEO) database under accession code GSE165823. The crystal structure of MEK1(C121S) reported in this paper has been deposited in the Protein Data Bank (PDB, ID code 7F2X). The structural data of wild-type MEK1 (PDB ID:, 3EQC, 3EQD, 3EQF, 3EQG, 3EQH, 3EQI, 3SLS, 3W8Q, 3ZLS, 3ZLX, 3ZLY, 3ZLW, 3ZM4, 5BX0, and 5HZE), MEK1-KSR2 complex (PDB ID: 2Y4I), MEK1-BRaf complex (PDB ID: 4MNE), and PKA (PDB ID: 1ATP and 4HPU) were obtained from the PDB database. All other data are available in the article and its Supplementary Information/Source Data file, or from the corresponding author upon reasonable request. Source data are provided with this paper.

## Source data

Source Data
