# Supplementary material for: Qualitative differences in disease-associated MEK mutants reveal molecular signatures and aberrant signaling-crosstalk in cancer
Source: Nat Commun. 2022 Jul 13;13:4063. doi: 10.1038/s41467-022-31690-w (PMC9279491; doi:10.1038/s41467-022-31690-w)
Supplement: Supplementary file 1 — Supplementary Information [file 41467_2022_31690_MOESM1_ESM.pdf]

Supplementary Information

Qualitative differences in disease-associated MEK mutants reveal molecular signatures and aberrant signaling-crosstalk in cancer

Yuji Kubota[1], Yuko Fujioka[2,3], Ashwini Patil[4], Yusuke Takagi[1], Daisuke Matsubara[5], Masatomi Iijima[2], Isao Momose[2], Ryosuke Naka[1], Kenta Nakai[4], Nobuo N. Noda[2,3] and Mutsuhiro Takekawa[1,]*

[1]Division of Cell Signaling and Molecular Medicine, Institute of Medical Science, The University of Tokyo, Minato-ku, Tokyo 108-8639, Japan

[2]Institute of Microbial Chemistry, Microbial Chemistry Research Foundation, Shinagawa-ku, Tokyo 141-0021, Japan

[3]Division of Biological Molecular Mechanisms, Institute for Genetic Medicine, Hokkaido University, Sapporo 060-0815, Japan

[4]Laboratory of functional analysis in silico, Human Genome Center, Institute of Medical Science, The University of Tokyo, Minato-ku, Tokyo 108-8639, Japan

[5]Molecular Pathology Laboratory, Institute of Medical Science, The University of Tokyo, Minato-ku, Tokyo 108-8639, Japan

*Correspondence to: takekawa@ims.u-tokyo.ac.jp

Supplementary Tables 1 and 2

Supplementary Figures 1-9

Supplementary Table 1: Data collection and refinement statistics (X-ray crystallography).

	MEK1 C121S
**Data collection**	
Space group	*F*222
Cell dimensions	
a, *b*, *c* (Å)	49.50, 156.50, 179.16
α, β, γ (°)	90.00, 90.00, 90.00
Resolution (Å)	50.00-2.01 (2.13-2.01) *
R_{sym} or R_{merge}	0.198 (1.207)
$I / \sigma I$	5.59 (1.15)
Completeness (%)	97.8 (95.0)
Redundancy	6.8 (6.6)
**Refinement**	
Resolution (Å)	44.79-2.01
No. reflections	23061
R_{work} / R_{free}	0.215/0.232
No. atoms	
Protein	2354
Ligand/ion	31
Water	53
B-factors (Å^2/Da)	
Protein	60.8
Ligand/ion	53.3
Water	51.9
R.m.s. deviations	
Bond lengths (Å)	0.002
Bond angles (°)	0.583

*One crystal was used for data collection. *Values in parentheses are for highest-resolution shell.

Supplementary Table 2: Primers for qRT-PCR.

Gene	Sequence
GAPDH-F	5’- CTATAAATTGAGCCCGCAGCC-3’
GAPDH-R	5’-ACCAAATCCGTTGACTCCGA-3’
DMRT1-F	5’- CAGGAAACCAGTGGCAGATGA-3’
DMRT1-R	5’- AGAGGGAGGCGGGTAGTAAG-3’
CEMIP-F	5’- CCAGACTAGCTACCACTCCGC-3’
CEMIP-R	5’- AGTGTGCTCCCTCTGGGTCT-3’
TFPI2-F	5’- GCCAACAGGAAATAACGCGG-3’
TFPI2-R	5’- AAATTGTTGGCGTTGCCCTC-3’
COL14A1-F	5’- AAGGCAATGAACGCATCAGCTA-3’
COL14A1-R	5’- CCCATGATGTGGAGCAAACAA-3’
c10orf10-F	5’- ACCTCCTCAGCTCCAGGTTG-3’
c10orf10-R	5’- TCCCGAATTGTGGGCAGATG-3’
BMP2-F	5’- CTGCGGTCTCCTAAAGGTCG-3’
BMP2-R	5’- CAACTCGAACTCGCTCAGGA-3’
IL11-F	5’- ACATGAACTGTGTTTGCCGC-3’
IL11-R	5’- AGCTGGGAATTTGTCCCTCAG-3’
CRLF1-F	5’- GCGAGAGGTCCTGCCAGATA-3’
CRLF1-R	5’- GAGCTGTTATGGCCGTTGGA-3’
THBD-F	5’- TCCTCTGCGAGTTCCACTTC-3’
THBD-R	5’- GCCGTAGGTGATCGAGACG-3’
GDF15-F	5’- TACTCACGCCAGAAGTGCGG-3’
GDF15-R	5’- CTTGCAAGGCTGAGCTGACG-3’
PHLDA1-F	5’- GGGCAAGACAAGGTTTTGAGG -3’
PHLDA1-R	5’- AACTACTTGATCTGGTGCGGG -3’
PHLDA2-F	5’- CGACAGCCTCTTCCAGCTAT -3’
PHLDA2-R	5’- AGTCGATCTCCTTGTGGTCG -3’
PHLDA3-F	5’- GCCTCTGCCAGATGCCTCC -3’
PHLDA3-R	5’- GGCACATCCCGCGAGCTGCC -3’
c11orf96-F	5’- ATGCCCTTGTCCGATGGTTT -3’
c11orf96-R	5’- ACGGCCACGCCTAACAATTA -3’
TNFRSF12A-F	5’- CTGCTTTGGCCCATCCTTG-3’
TNFRSF12A-R	5’- CCTCTATGGGGGTGGTGAAC-3’
SLC20A1-F	5’- CGCTCTCTGCGTGGTTCTTC-3’
SLC20A1-R	5’- GTTCATCCAGGGAAAAGGGAGG-3’
IL4R-F	5’- GGGGCGCGCAGATAATTAAAG -3’
IL4R-R	5’- GAACAGGAGCCCAGAGCAAA -3’
CD44-F	5’- AGCACAATCCAGGCAACTCC-3’
CD44-R	5’- TGTCCCTGTTGTCGAATGGG-3’
TM4SF1-F	5’- GCAAACGATGTGCGATGCTT-3’
TM4SF1-R	5’- ACTCGGACCATGTGGAGGTA-3’
TM4SF19-F	5’- TCGAAGCGTGCTTACTGCTC-3’
TM4SF19-R	5’- CGAACGGTCATACAGATAATTCC-3’
EMP1-F	5’- GTGCTGGCTGTGCATTCTTG-3’
EMP1-R	5’- CCGTGGTGATACTGCGTTCC-3’
SEMA7-F	5’- CACCATCCGGAAGCAGGAATA-3’

SEMA7-R	5'- TGCACGATGGTGGCTTTGA-3'
CD3D-F	5'- CATGGGTAGAGGGAACGGTG-3'
CD3D-R	5'- ACAGCTCTGGCACATTCGAT-3'
ERRFI1-F	5'- GGAGCAGTCGCAGTGAGTTT -3'
ERRFI1-R	5'- ATTTGGAAGCATGCCCAAGTG -3'
GADD45B-F	5'- TTGTCTCCTGGTCACGAACC-3'
GADD45B-R	5'- TGTGGCAGCAACTCAACAGA-3'
IER3-F	5'- GCCGCCTTCTAACTGTGACT-3'
IER3-R	5'- CGTCTCCGCTGTAGTGTTCT-3'
c8orf4-F	5'- TGGCTACCACTTCGACACAG-3'
c8orf4-R	5'- ATCTTGGCTCTCTCCTCTGC-3'
PLAUR-F	5'- ATTCCCGAAGCCGTTACCTC -3'
PLAUR-R	5'- AAGCTCCAGGACTTCTTCACC -3'
MMP1-F	5'- GCGACTCTAGAAACACAAGAGC-3'
MMP1-R	5'- CAATCCTGTAGGTCAGATGTGTT-3'
MMP10-F	5'- GACAAAGAAGGTAAGGGCAGTG-3'
MMP10-R	5'- ACTTTTCTAGGTATTGCTGGGC-3'
IL13RA2-F	5'- TCACCACAAGGAATTCCAGAAAC-3'
IL13RA2-R	5'- TGCATGATCCAAGCCCTCAT-3'
TRIB1-F	5'- ACTACCTGCTGCTGCCCCTA-3'
TRIB1-R	5'- AATGGGAAACACCTTGCAGCG-3'
TRIB3-F	5'- GTCTTCGCTGACCGTGAGA-3'
TRIB3-R	5'- CAGTCAGCACGCAGGAGTC-3'
DUSP5-F	5'- TCCTGAGTGTTGCGTGGATG-3'
DUSP5-R	5'- GGCCACCCTGGTCATAAGC-3'
DUSP6-F	5'- CGTTCTACCTGGAAGGTGGC-3'
DUSP6-R	5'- CCATCCGAGTCTGTTGCACT-3'
PRR9-F	5'- AGATCAGGCTCTGCAAATCAC-3'
PRR9-R	5'- GGGCCTGCACTAGACACTTA-3'
EGR1-F	5'- CTGACCCGTTCGGATCCTTT-3'
EGR1-R	5'- AGGAAAAGACTCTGCGGTCA-3'
SPANXN3-F	5'- GATGAGATGCAAGAGGTACCAAA-3'
SPANXN3-R	5'- CCCTTCCTGAGGTAATACACAAA-3'
SPANXN4-F	5'- GGAAGAGCCAACTTCCAGCA-3'
SPANXN4-R	5'- TGCAGATTCTTCTTCTTCTTGTCA-3'
SPANXN5-F	5'- TGATGAGATGCAGGAGACACC-3'
SPANXN5-R	5'- TCTTCCTGTAGCACAACACTAATAC-3'
IRX4-F	5'- GGCTCCCCAGTTCTTGATGG-3'
IRX4-R	5'- TAGACCGGGCAGTAGACCG-3'
LINC00460-F	5'- CATGTTGCAGCTTTCCCACG-3'
LINC00460-R	5'- TCTTCTTTCCCACGCTCAGTC-3'
LINC00261-F	5'- TCACAGGAAATGGCATCAAG-3'
LINC00261-R	5'- TGCAGAATCCTGACACAGTG-3'
LINC00941-F	5'- AGAATCAGTCAGCAAGGAGG-3'
LINC00941-R	5'- ACTAGGATGGCCTGAATCTG-3'
LUCAT-F	5'- AACTCTTATGGGACCTTGGC-3'
LUCAT-R	5'- CAGAGCGAAACTCTGTAGCTC-3'
LINC00659-F	5'- CCCCTCTCTTGGGAGGTAGAG-3'
LINC00659-R	5'- CTGTCTCCACGGTGTCCAAG-3'

Supplementary figures

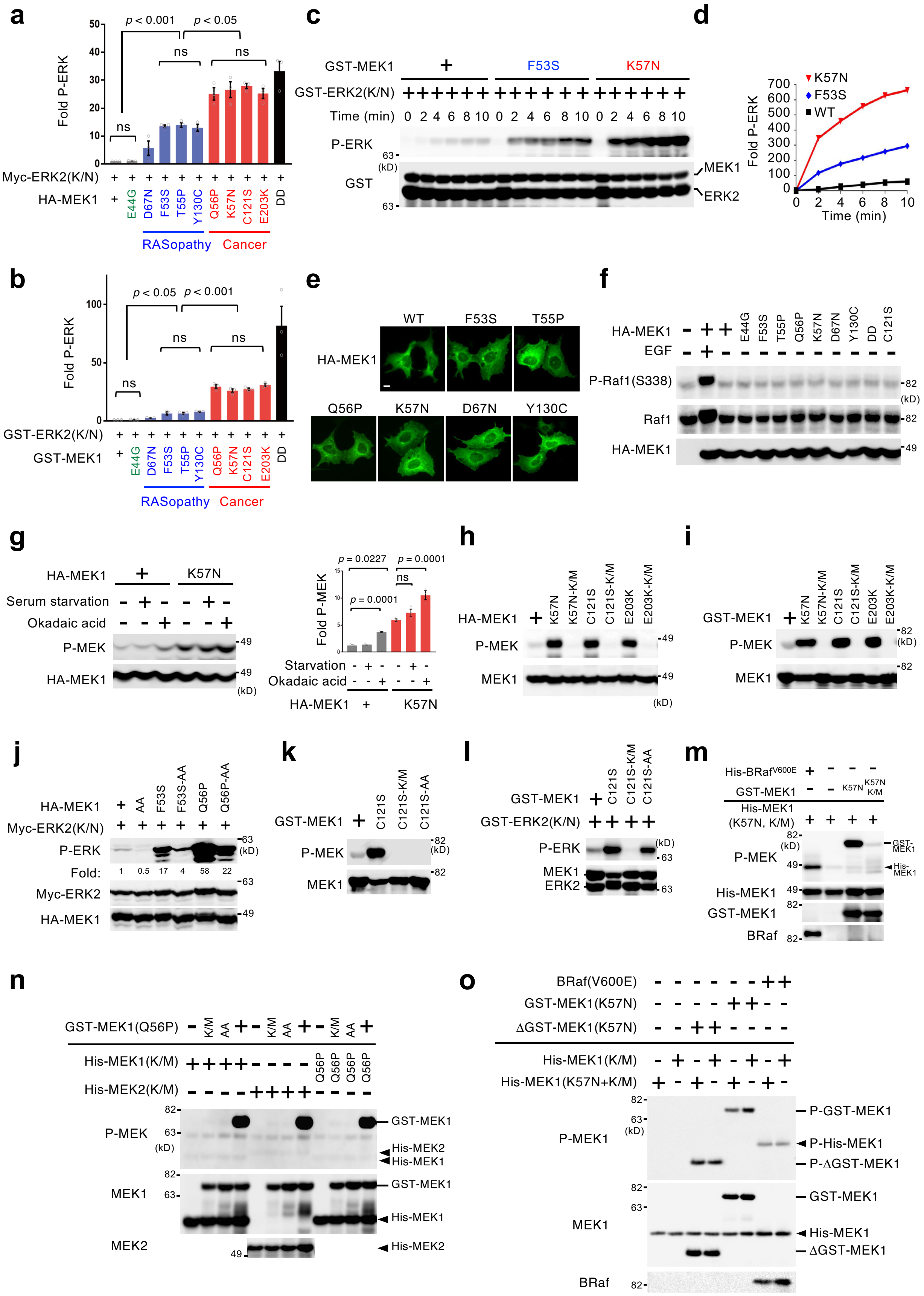

Supplementary Fig. 1 Cancer- and RASopathy-associated MEK1 mutations differentially modulate MEK1 activity through distinct molecular mechanisms.

a, **b**, In **a**, HEK293 cells were transfected with HA-MEK1 (WT or its indicated mutants) together with kinase-deficient Myc-ERK2(K/N) as described in Fig. 1b. In **b**, an in vitro kinase assay of purified recombinant GST-MEK1 proteins was performed using GST-ERK2(K/N) as a substrate as in Fig 1c. Phosphorylated ERK2 (P-ERK) was probed by immunoblotting. The mean intensity of the P-ERK bands from three independent experiments was quantified.

c, **d**, In vitro kinase assay of purified recombinant GST-MEK1 using GST-ERK2(K/N) as a substrate. In **c**, GST-MEK1-WT (+) or the indicated mutants were incubated with GST-ERK2(K/N) for the indicated times. Phosphorylated ERK2(K/N) (P-ERK) was detected by immunoblotting (top). Total GST-MEK1 and GST-ERK2(K/N) were also probed with an anti-GST Ab (bottom). **d**, The intensity of the P-ERK bands in **c** was quantified.

e, HA-MEK1 or its mutant derivatives were transiently expressed in HEK293 cells and were detected by immunofluorescent staining with an anti-HA Ab. Scale bar, 10 μm.

f, Disease-associated MEK mutations do not affect Raf-1 activity. HEK293 cells were transfected with HA-MEK1 or its mutant derivatives. The cell lysates were probed for the activating phosphorylation of Raf-1 at S338 by immunoblotting (top). Protein levels of Raf and MEK1 were also probed (middle and bottom). Where indicated, cells were stimulated with EGF as a positive control for Raf-1 activation.

g, HA-MEK1 (WT or K57N) expressed in HEK293 cells was immunoblotted with an anti-phospho-MEK1(S218/S222) Ab (P-MEK) (left). Where indicated, the cells were serum starved over night (serum starvation) or pretreated with a PP2A inhibitor (okadaic acid, 300 nM for 2 h). MEK1(K57N) autophosphorylation was detected under all the conditions tested. The mean intensity of the P-MEK bands from three independent experiments was quantified and is shown as a bar graph (right).

a, **b**, **g**, Data are mean ± SEM (n=3). P-values were assessed by a one-way ANOVA Tukey test. ns, not significant.

h, i, **k**, A kinase-inactivating K97M (K/M) mutation abrogates the phosphorylation of the cancer-associated MEK1 mutants. The activation-associated MEK1 phosphorylation at S218/S222 was detected by immunoblotting with an anti-phospho-MEK1 Ab (P-MEK1) (top). The total amounts of MEK1 were also probed (bottom). **h**, HEK293 cells were transfected with HA-MEK1 (WT or its indicated mutants). Cell lysates were analyzed by immunoblotting. **i**, **k**, The indicated GST-MEK1 proteins were expressed in *E. coli*, purified, and subjected to immunoblotting. In **k**, AA, S218A/S222A non-phosphorylatable mutant.

j, HEK293 cells were transiently transfected with HA-MEK1 (WT or the indicated mutants) and Myc-ERK2(K/N). P-ERK was detected by immunoblotting (top). Expression levels of the proteins are shown in the lower panels.

l, In vitro kinase assay. The purified GST-MEK1 proteins were incubated with GST-ERK2(K/N), followed by immunoblotting with an anti-phospho-ERK Ab (P-ERK).

m, MEK1 autophosphorylation does not occur in *trans*. The indicated recombinant GST-MEK1 mutants or His-BRaf^{V600E} (as a positive control) were incubated with kinase-dead His-MEK1(K57N, K/M). The reaction mixture was separated by SDS-PAGE and probed with anti-P-MEK Ab. Total protein levels were also probed (the lower panels)

n, Autophosphorylation of the MEK1 mutants does not occur in *trans*. The indicated recombinant GST-MEK1(Q56P) proteins [highly active, inactive (K/M), or moderately active (AA) enzymes] were incubated with kinase-deficient His-MEK1(K/M), His-MEK1(K/M, Q56P), or His-MEK2(K/M) proteins (substrates) in vitro. The reaction mixtures were separated by SDS-PAGE and probed for phosphorylated MEK (top) and total MEK (middle and bottom).

o, The indicated recombinant MEK1(K57N) or BRaf^{V600E} proteins (enzymes) were incubated with kinase-deficient His-MEK1(K/M) or His-MEK1(K57N, K/M) (substrates). Where indicated, MEK1(K57N) protein was liberated from GST-tag by cleavage with PreScission protease, and the resulting GST-free MEK1(K57N) [ΔGST-MEK1(K57N)] was used as an enzyme. The reaction mixtures were then separated by SDS-PAGE and probed for phosphorylated MEK1 (top), total MEK1 (middle), and total BRaf^{V600E} (bottom). BRaf^{V600E}-mediated phosphorylation of His-MEK1(K/M) or His-MEK1(K57N, K/M) is shown as a positive control.

Source data are provided as a Source Data file.

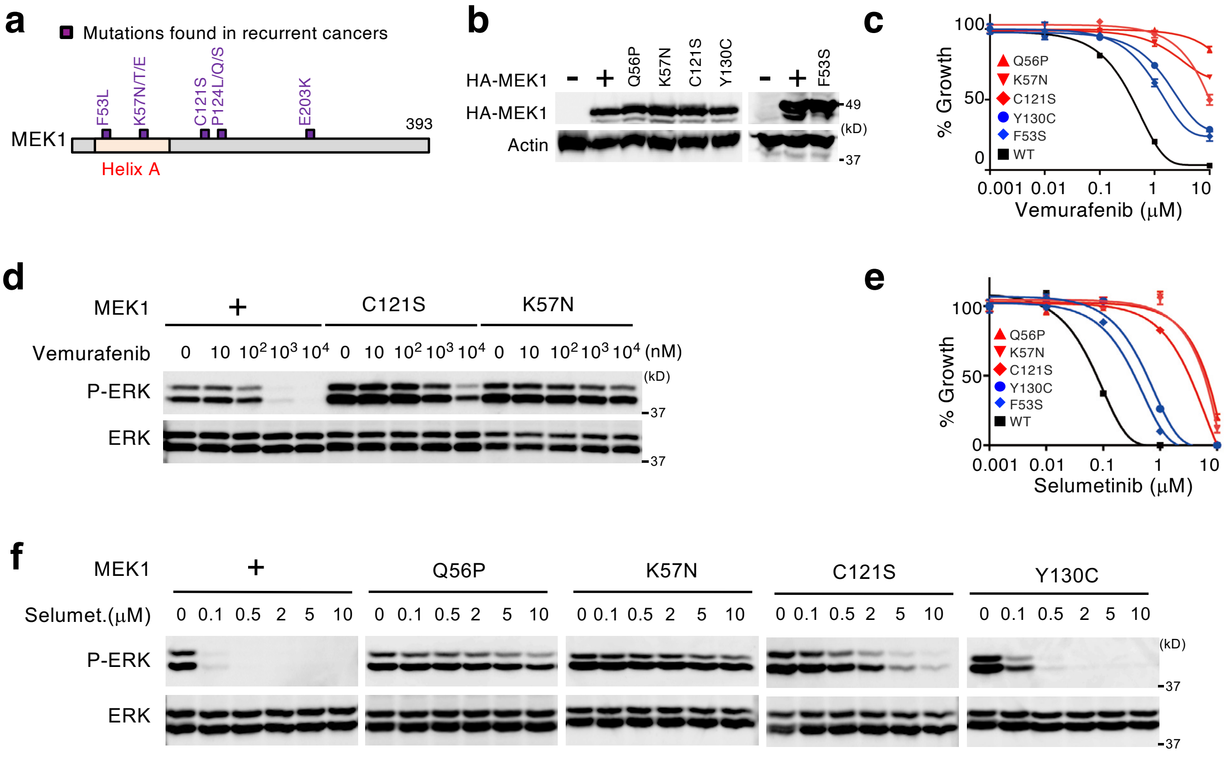

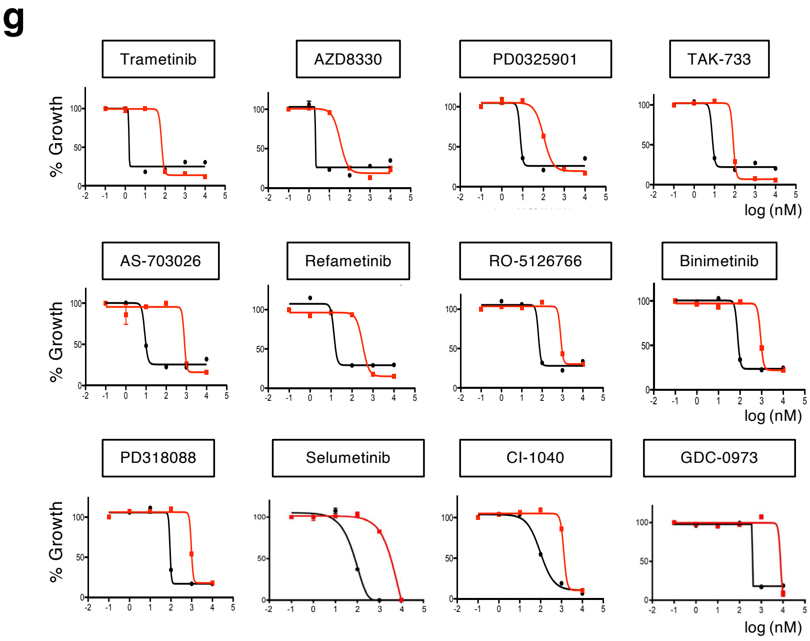

h

	MEK1 GI50 (nM)	MEK1 (C121S) GI50 (nM)	GI50 Ratio (C121S/WT)
Trametinib	1.5	65.5	43.0
AZD8330	2.1	34.2	16.6
PD0325901	7.6	103.7	13.6
TAK-733	7.6	83.5	10.9
AS-703026	8.7	788.9	90.7
Refametinib	13.8	319.9	23.2
RO-5126766	65.7	805.3	12.3
Binimetinib	76.9	896.4	11.7
PD318088	90.0	964.2	10.7
Selumetinib	94.3	1341	14.2
CI-1040	97.0	1285	13.3
GDC-0973	281.9	7571	26.9

Supplementary Fig. 2 Disease-associated MEK1 mutations confer resistance to ERK pathway-targeted therapeutics.

a, Schematic diagram of MEK1 mutations in recurrent cancers.

b-f, In **b**, $BRaf^{V600E}$-positive A375 cells were infected with a retroviral vector encoding HA-MEK1 or its indicated mutants (cancer-associated Q56P, K57N, or C121S, or RASopathy-associated Y130C or F53S). Comparable expression levels of the HA-MEK1 proteins in individual cells were confirmed by immunoblotting. Actin, loading control. **c** and **e**, Growth inhibition assays. A375 cells stably expressing the indicated MEK1 mutants were cultured in various concentrations of vemurafenib (**c**) or a MEK inhibitor (selumetinib) (**e**) for 4 days. The rate of cell proliferation (%Growth) was determined by CCK8 assay. Error bars, SEM (n=3). **d** and **f**, A375 cells expressing the indicated mutants were treated with various concentrations of vemurafenib (**d**) or selumetinib (**f**) for 1 h, and the cell lysates were probed for phosphorylated ERK (P-ERK) (top) and total ERK (bottom).

g, **h**, MEK1-C121S mutation confers resistance to growth inhibition induced by all the allosteric MEK inhibitors tested. **g**, A375 cells expressing HA-MEK1 (WT or C121S) were cultured in the presence of various concentrations of the MEK inhibitors. The rate of cell proliferation (% Growth) was determined as in **e**. **h**, The half maximal growth inhibitory concentrations (GI50) of individual inhibitors are shown.

Source data are provided as a Source Data file.

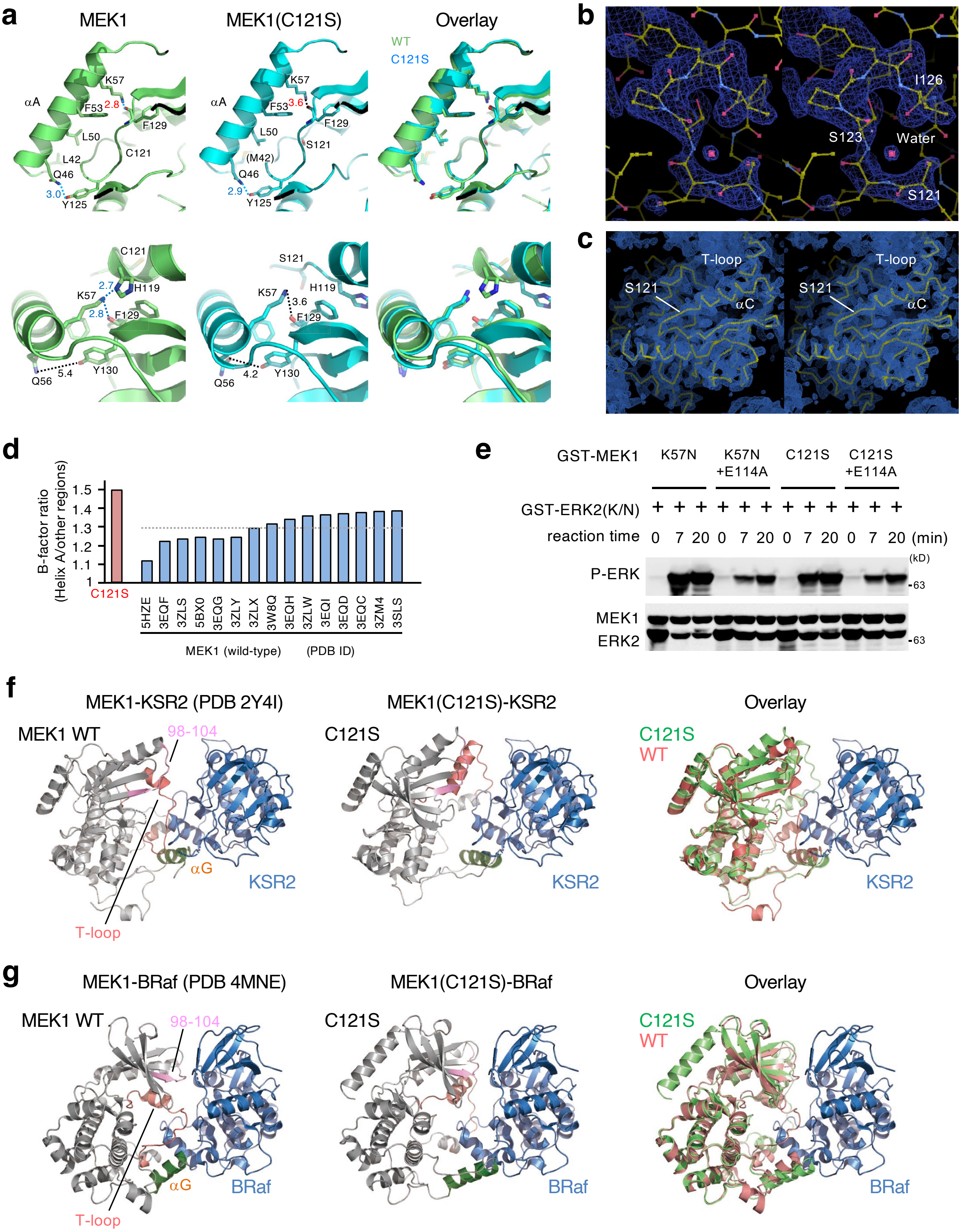
a
MEK1
MEK1(C121S)
Overlay
WT
C121S
αA
K57
F53
2.8
F129
L50
C121
L42
Q46
3.0
Y125
K57
F53
3.6
F129
L50
S121
(M42)
Q46
2.9
Y125
C121
2.7
H119
K57
2.8
F129
Q56
5.4
Y130
S121
K57
H119
3.6
F129
Q56
4.2
Y130
b
I126
S123
Water
S121
c
T-loop
S121
αC
d
B-factor ratio
(Helix A/other regions)
C121S
5HZE
3EQF
3ZLS
5BX0
3EQG
3ZLY
3ZLX
3W8Q
3EQH
3ZLW
3EQI
3EQD
3EQC
3ZM4
3SLS
MEK1 (wild-type)
(PDB ID)
e
GST-MEK1
K57N
K57N +E114A
C121S
C121S +E114A
GST-ERK2(K/N)
reaction time
0 7 20 0 7 20 0 7 20 0 7 20 (min)
(kD)
P-ERK
63
MEK1
ERK2
63
f
MEK1-KSR2 (PDB 2Y4I)
MEK1(C121S)-KSR2
Overlay
MEK1 WT
98-104
αG
KSR2
T-loop
C121S
KSR2
C121S
WT
KSR2
g
MEK1-BRaf (PDB 4MNE)
MEK1(C121S)-BRaf
Overlay
MEK1 WT
98-104
αG
BRaf
T-loop
C121S
BRaf
C121S
WT
BRaf

Supplementary Fig. 3 Structural and mutational analyses of cancer-associated MEK1(C121S).

a, Close-up views of the inhibitory A-helixes (αA). The A-helixes and their adjacent areas of MEK1 WT (PDB: 3SLS) (left) and C121S (middle) are shown in two orientations related by a 90˚ rotation along the horizontal axis (top and bottom rows). Superpositions of these two structures are also shown (right). Nitrogen and oxygen atoms are colored blue and red, respectively. The numbers beside the dotted lines represent distances (Å). An H-bond was considered when the donor-acceptor distance was less than 3.5 Å and the angle formed by donor-hydrogen-acceptor was greater than 120˚.

b, **c**, Stereo-views of the unbiased composite omit map (**c**) and the polder map omitting residues 121-126 (**b**) of MEK1(C121S) calculated using the program Phenix. The maps were shown with the Cα trances (**c**) and the stick models of the final model of MEK1 C121S (**b**) using the program COOT.

d, C121S mutation increases the flexibility of the helix A. The B-factor ratio of the helix A to other regions in MEK1 is shown as a bar graph. The dotted line indicates the average across 15 WT MEK1 structures reported in the PDB.

e, E144 residue on the αC helix is not essential for the kinase activity of the MEK1 mutants. The indicated GST-MEK1 proteins were incubated with GST-ERK2(K/N). ERK phosphorylation was immunoblotted as in Fig. 1c.

f, **g**, The overall structure of MEK1(C121S) is superimposed on that of the MEK1-KSR2 complex (PDB 2Y4I) (**f**) or the MEK1-BRaf complex (PDB 4MNE) (**g**). The conformation and orientation of the MEK1 T-loop and αG helix, which form an interaction surface with KSR2 and BRaf [4,5], are altered in MEK1(C121S) compared with MEK1, suggesting that C121S mutation compromises the interactions of MEK1 with KSR and BRaf at least to some extent.

Source data are provided as a Source Data file.

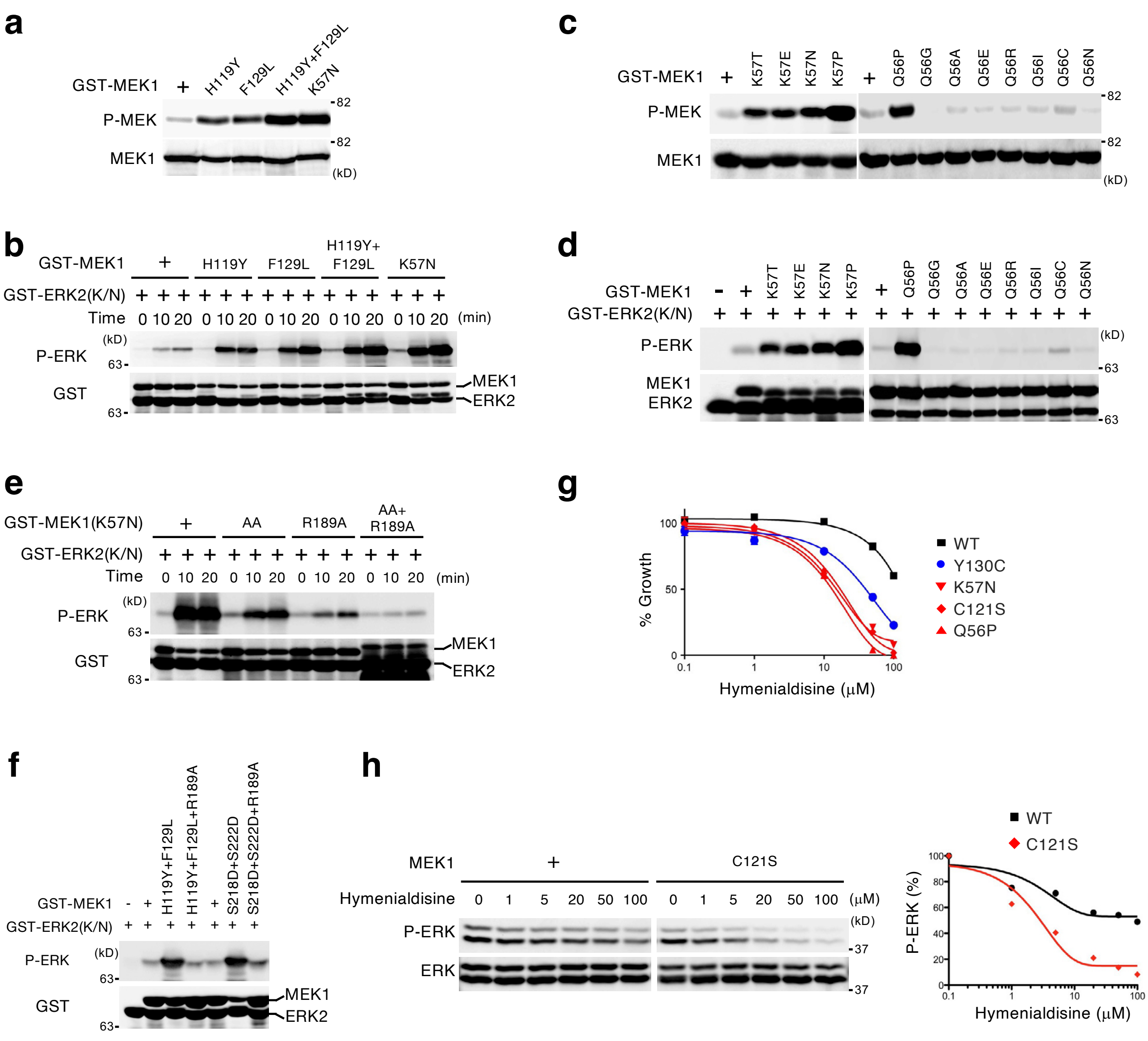

Supplementary Fig. 4 Disruption of the H-Bonds of K57 to H119 and F129 is critical for the acquisition of the abnormal autophosphorylation activity of cancer-associated MEK1 mutants.

a-d, Disruption of the interdomain H-bonds is critical for the autophosphorylating activity of cancer-associated MEK1 mutants. GST-MEK1 WT or the indicated mutants were expressed in, and purified from bacteria, and their phosphorylation states (**a**, **c**) and kinase activities (**b, d**) were assessed as in Fig. 1g and h, respectively.

e, **f**, R189 is involved in the enhanced kinase activity of the MEK1 mutants. The indicated GST-MEK1 proteins were incubated with GST-ERK2(K/N). ERK phosphorylation was immunoblotted as in **b**. AA, S218A/S222A.

g, **h**, The ATP-competitive inhibitor, hymenialdisine, preferentially inhibits the disease-associated, hyperactive MEK1 mutants. **g**, BRaf^{V600E}-positive A375 cells stably expressing HA-MEK1 or its mutants (cancer-associated Q56P, K57N, or C121S, or RASopathy-associated Y130C) were cultured in various concentrations of hymenialdisine for 4 days, and the rate of cell proliferation was assessed by CCK-8 assay. Error bars, SEM (n=3). **h**, A375 cells expressing HA-MEK1 (WT or C121S) were treated with hymenialdisine for 1 h. The cell lysates were probed for ERK phosphorylation (left). The intensity of the P-ERK bands was quantified (right graph).

Source data are provided as a Source Data file.

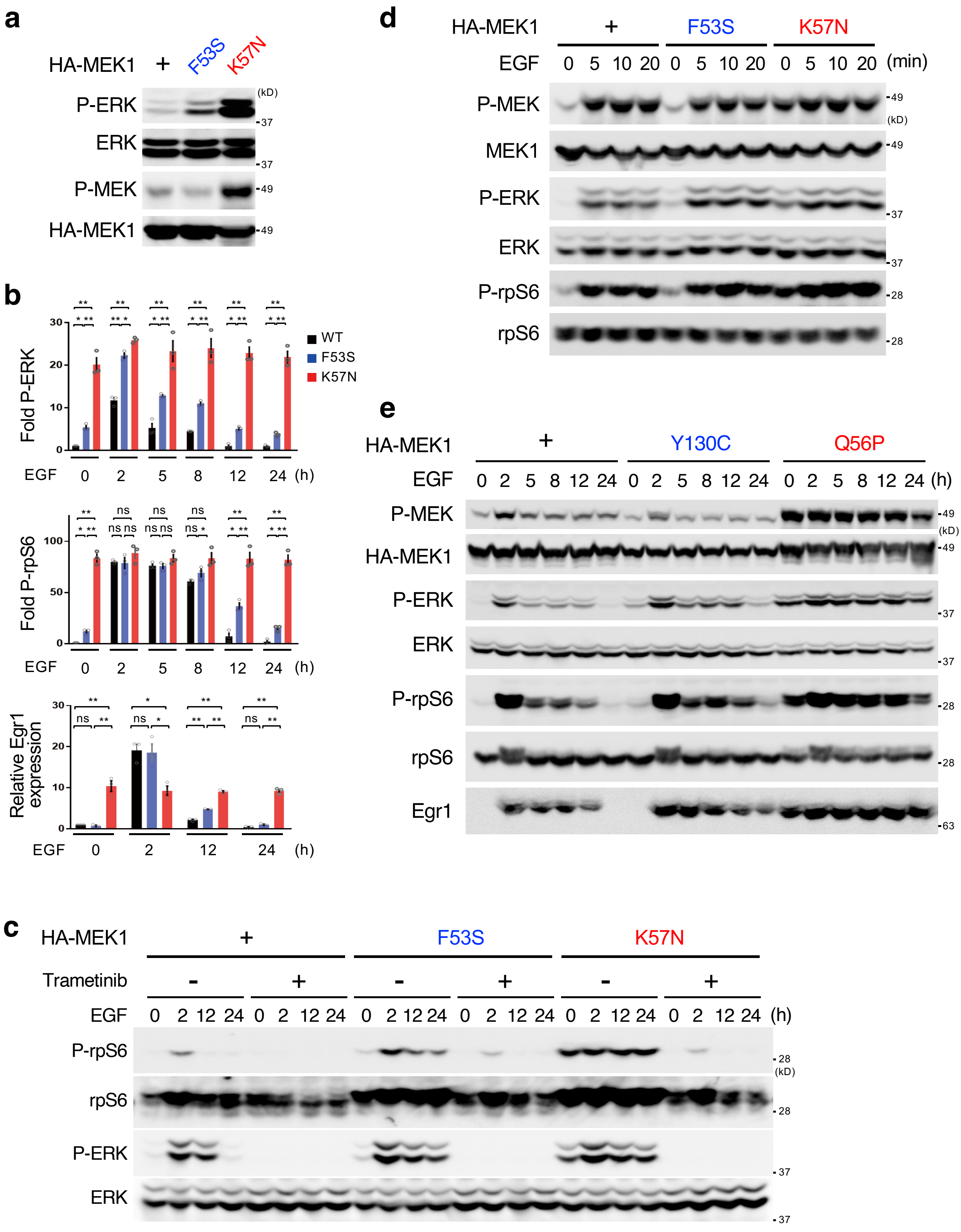
a
HA-MEK1 + F53S K57N
P-ERK
ERK
P-MEK
HA-MEK1
(kD)
37
37
49
49
b
Fold P-ERK
WT
F53S
K57N
EGF 0 2 5 8 12 24 (h)
Fold P-rpS6
EGF 0 2 5 8 12 24 (h)
Relative Egr1 expression
EGF 0 2 12 24 (h)
ns
c
HA-MEK1 + F53S K57N
Trametinib - + - + - +
EGF 0 2 12 24 0 2 12 24 0 2 12 24 0 2 12 24 0 2 12 24 0 2 12 24 (h)
P-rpS6
rpS6
P-ERK
ERK
28
(kD)
28
37
37
d
HA-MEK1 + F53S K57N
EGF 0 5 10 20 0 5 10 20 0 5 10 20 (min)
P-MEK
MEK1
P-ERK
ERK
P-rpS6
rpS6
49
(kD)
49
37
37
28
28
e
HA-MEK1 + Y130C Q56P
EGF 0 2 5 8 12 24 0 2 5 8 12 24 0 2 5 8 12 24 (h)
P-MEK
HA-MEK1
P-ERK
ERK
P-rpS6
rpS6
Egr1
49
(kD)
49
37
37
28
28
63

Supplementary Fig. 5 MEK mutations alter the spatiotemporal dynamics of ERK signaling.

a, Phosphorylation states of ERK in HEK293 cells stably expressing HA-MEK1(WT, F53S, or K57N). HA-MEK1(WT, F53S, or K57N) was stably expressed in HEK293 cells. The cell lysates were immunoblotted for the expression levels and the phosphorylation states of HA-MEK1 and endogenous ERK.

b, HEK293 cells stably expressing HA-MEK1(WT, F53S, or K57N) were treated with EGF for the indicated times. The levels of phosphorylated ERK1/2 (P-ERK), phsophorylated-rpS6 (P-rpS6), and Egr1 expression were analyzed by immunoblotting using appropriate Abs as in Fig. 4a. Data represent the averages and SEMs of three independent experiments. p-values were assessed by a one-way ANOVA Tukey test (n=3). *$p < 0.05$, **$p < 0.01$.

c, EGF-induced rpS6 phsophorylation is mainly mediated by the ERK pathway. The indicated cells were stimulated with EGF for the indicated times in the presence or absence of the MEK inhibitor (trametinib). The phosphorylation levels of rpS6 and ERK were analyzed by immunoblotting. The expression levels of ERK1/2 and rpS6 in cell lysates are also shown.

d, The indicated cells were treated with EGF for relatively short periods of time (5-20 min) as indicated. The levels of phosphorylated ERK1/2 (P-ERK), phsophorylated rpS6 (P-rpS6), and Egr1 expression were analyzed by immunoblotting using appropriate Abs.

e, HEK293 cells stably expressing HA-MEK1 (WT, Y130C, or Q56P) were treated with EGF for the indicated times. The phosphorylation levels of MEK1, ERK, and rpS6 were analyzed by immunoblotting using appropriate phospho-specific Abs. The expression levels of HA-MEK1, ERK1/2, rpS6, and Egr1 in cell lysates are also shown.

Source data are provided as a Source Data file.

a

Group1 (linear)		Group2 (sigmoidal-A)	Group3 (sigmoidal-B)		Group4 (bell-shaped)		Group5 (reverse-linear)	
DMRT1	MMP2	AP1S2	EGR1	TFPI2	COL14A1	OPRM1	BMF	IGFBP5
DUSP2	FOXA1	C20orf151	JUNB	GDF15	CACNA1D	GRM6	BCL11B	BCL2L10
SPRY2	ADAM10	C20orf160	FOS	PHLDA1	CACNG1	MC3R	RARB	NPNT
SPRY4	SBSN	XLOC_010216	FOSB	PHLDA2	GRIA4	PTGIR	TRPV6	SOX11

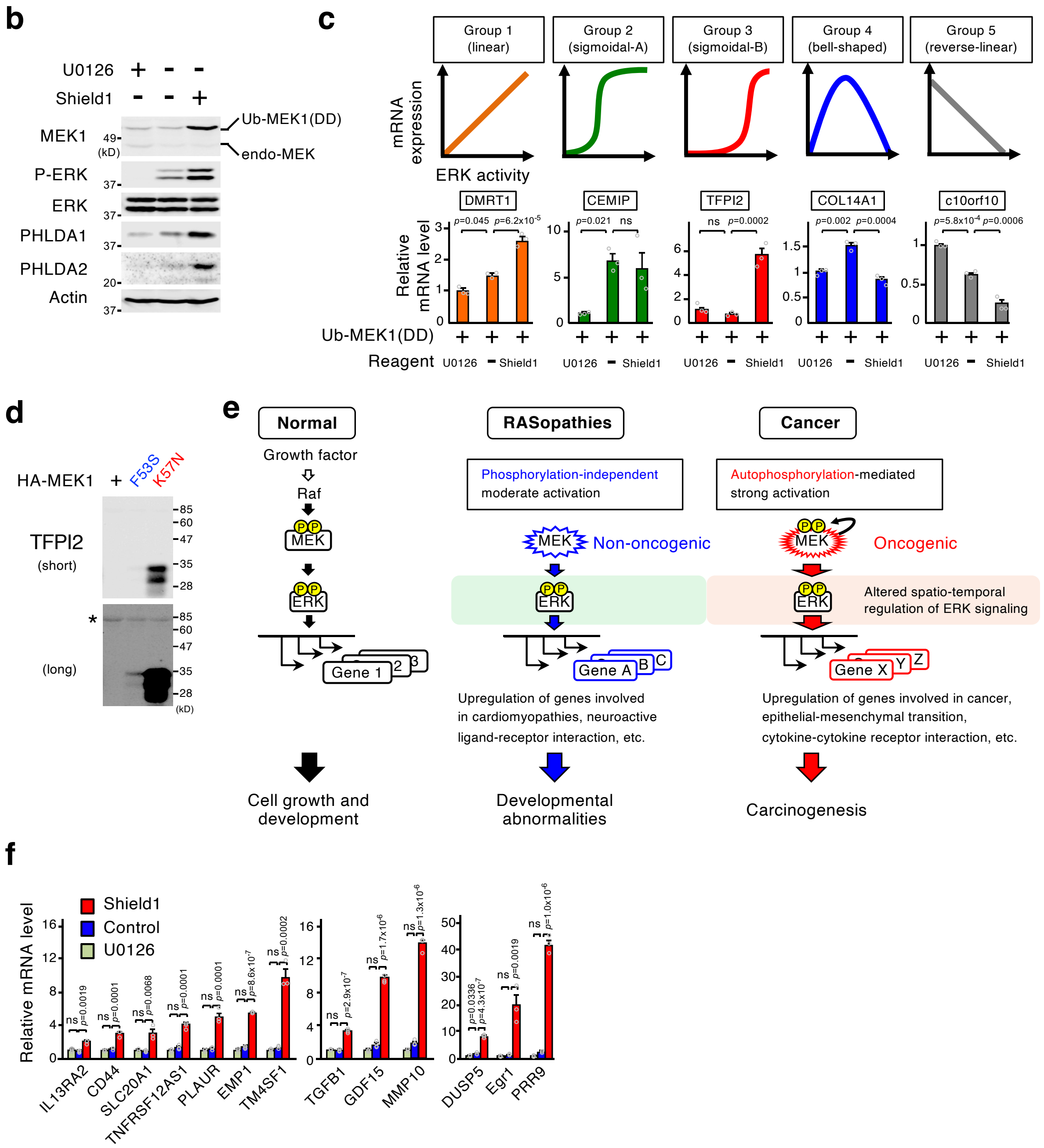

Supplementary Fig. 6 MEK1 mutations generate distinct gene expression patterns that are related to the degree of aberrant ERK activity.

a, A list of representative genes in each group.

b, **c**, Reconstitution of the ERK activity-dependent gene expression patterns using drug-induced expression of a constitutively active MEK1(DD) mutant. MEK1(DD), fused to the ubiquitination (Ub)-mediated destabilization domain derived from the ProteoTuner system (Clontech) [Ub-MEK1(DD)], was stably transfected into HEK293 cells [HEK293-Ub-MEK1(DD) cells]. Since the Ub-MEK1(DD) protein is persistently ubiquitinated and rapidly degraded in these cells, it is expressed at a low level and, therefore, ERK activity is only moderately elevated. However, when the cells are treated with the cell-permeable ligand Shield1, Shield1 binds to the destabilization domain and protects it from Ub-mediated degradation, thereby inducing Ub-MEK1(DD) accumulation and consequent strong ERK activity. **b**, HEK293-Ub-MEK1(DD) cells were treated with Shield1 (50 nM for 24 h) or U0126 (20 μM for 6 h), or were left untreated (-) to reconstitute high, low, and moderate ERK activities in the cells. The indicated proteins were detected by immunoblotting. Actin, a loading control. **c**, Schematic representation of the gene expression patterns in the HEK293-Ub-MEK1(DD) cells treated with Shield1 or U0126 as in **b** (upper). The mRNA expression of a representative gene in each group was assessed by qRT-PCR using specific primers (lower).

d, Western blot analysis of TFPI2 expression in cell culture supernatants. The TFPI2 blot shown in Fig. 5g (short exposure) was exposed for a longer time. A non-specific background band of immunoblotting is shown as a loading control (asterisk).

e, A schematic model of abnormal ERK signaling induced by the disease-associated MEK1 mutations. RASopathy-associated mutations (e.g., F53S) moderately increase the basal kinase activity of MEK1 in a phosphorylation independent manner. Similarly, cancer-associated MEK1 mutants (e.g., K57N) are also moderately active even without T-loop phosphorylation. In addition, the cancer-associated mutants also acquire the ability to autophosphorylate their own T-loops, thereby exhibiting strong kinase activity. Moreover, these two types of MEK1 mutations differentially alter the spatio-temporal properties of ERK signaling. The RASopathy-associated mutations increase the magnitude and duration of growth factor-induced ERK activity and lead to the prolonged nuclear accumulation of ERK, whereas the cancer-associated mutations elicit persistent activation and nuclear translocation of ERK even without stimulation. Importantly, these two different spatio-temporal alterations in ERK signaling dynamics generate distinct gene expression patterns in cells that are related to the pathophysiology of RASopathies and cancers. As a result, the RASopathy- and cancer-associated MEK1 mutations can lead to the development of distinct clinical manifestations (developmental abnormalities and carcinogenesis, respectively), even though the same MEK1 gene is a target for mutation in both diseases.

f, Reconstitution of the sigmoidal-B pattern of gene expression using the drug-induced MEK1(DD) expression system. HEK293-Ub-MEK1(DD) cells were treated with U0126 or Shield1 as in **b**. The expression levels of the indicated mRNAs were analyzed using qRT-PCR.

c, **f**, Data are mean ± SEM from three independent experiments. P-values were assessed using one-way ANOVA followed by Tukey's multiple comparisons test. ns, not significant.

Source data are provided as a Source Data file.

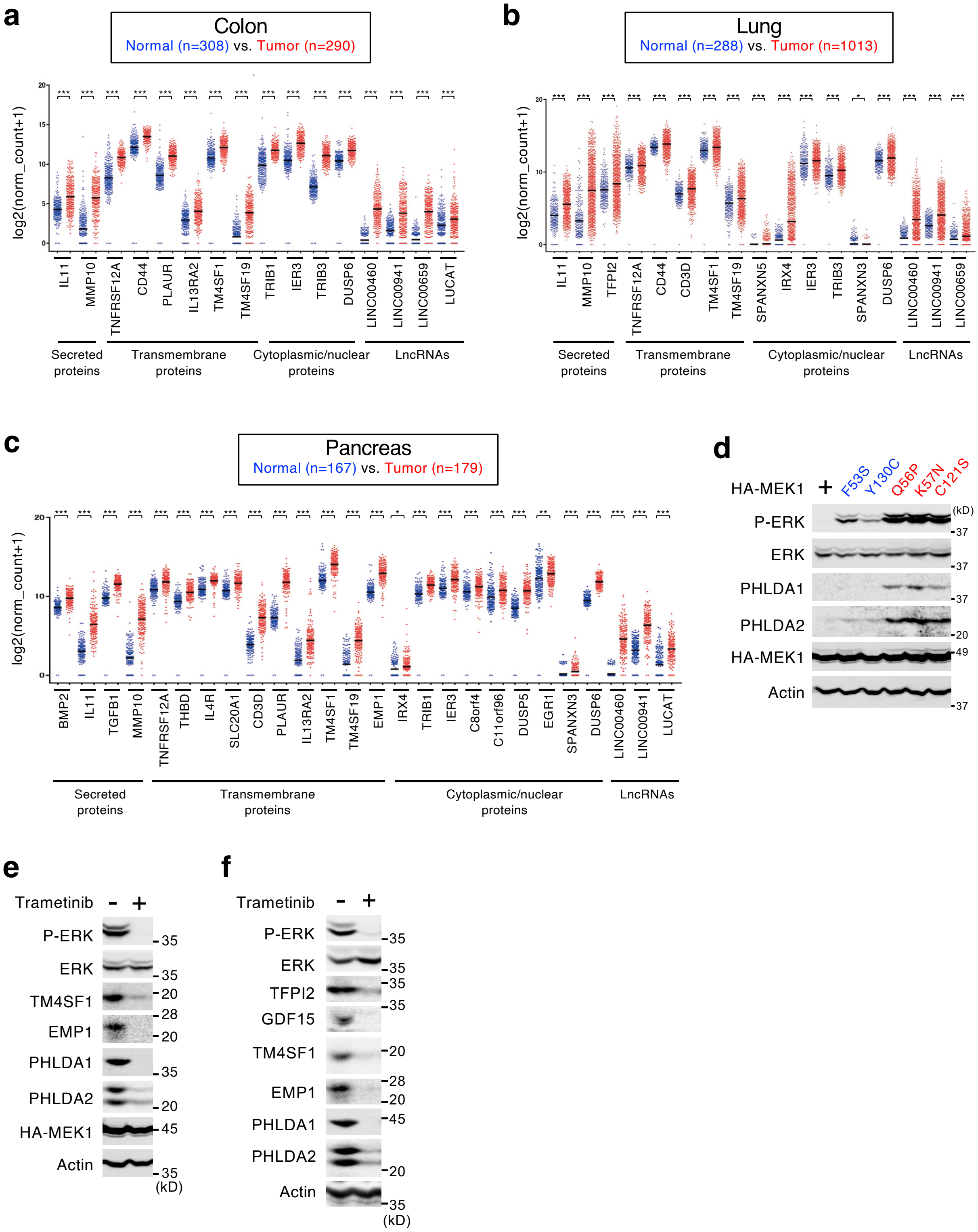

Supplementary Fig. 7 Genes upregulated by oncogenic MEK1(K57N) serve as cancer signature genes. **a-c**, Analyses of mRNA expression levels of the sigmoidal-B group of genes in normal vs. various cancer tissues based on the TCGA and GEO datasets. Each horizontal bar represents the mean. P-values were determined using two-tailed Student t-test. *, $p < 0.05$; **, $p < 0.01$; ***, $p < 0.001$.

d, The expression levels of PHLDA1/2 proteins in HEK293 cells expressing HA-MEK1(WT, F53S, Y130C, Q56P, K57N, or C121S) were assessed by immunoblotting.

e, **f**, The expression levels of the indicated proteins in K57N cells (**e**) and H1299 cells (**f**) were assessed by immunoblotting with the appropriate Abs. Where indicated, the cells were pretreated with the MEK inhibitor trametinib for 24 h. Source data are provided as a Source Data file.

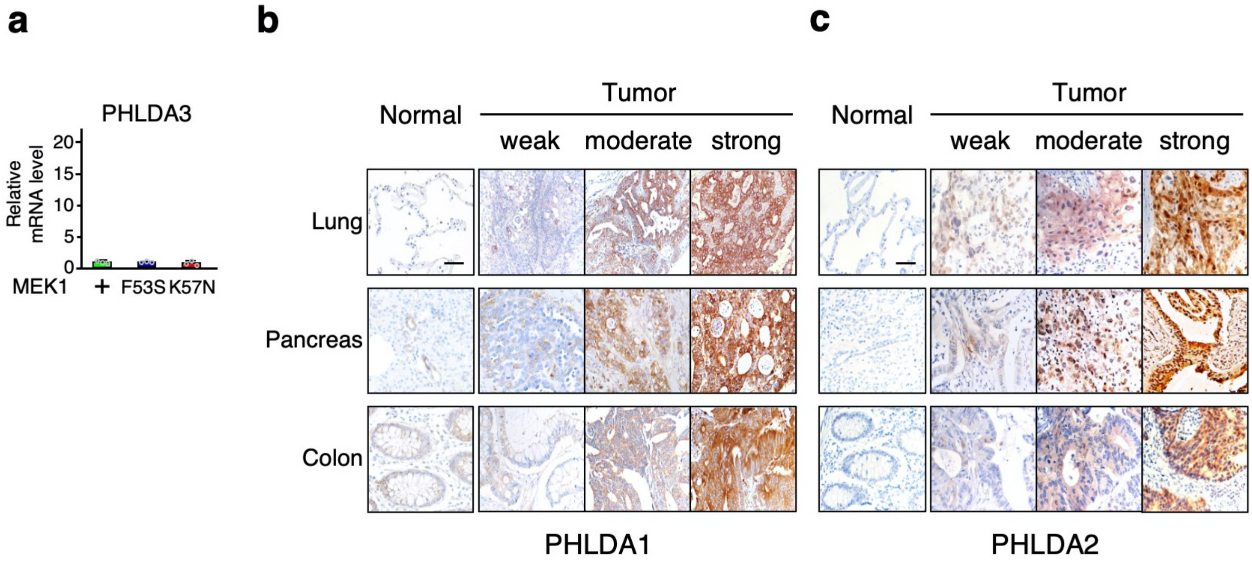

Supplementary Fig. 8 Oncogenic ERK signaling simultaneously upregulates PHLDA1 and PHLDA2.

a, PHLDA3 mRNA expression level in HEK293 cells stably expressing MEK1(WT, F53S, or K57N) were analyzed using qRT-PCR. Error bars, SEM (n=3).

b, **c**, Immunohistochemical staining for PHLDA1 (**b**) and PHLDA2 (**c**) in normal and tumor tissues of lung, pancreas, and colon. Tissue samples were immunostained with DAB and counterstained with hematoxylin. Representative images of weak, moderate, and strong staining are shown. Scale bars, 60 µm.

Source data are provided as a Source Data file.

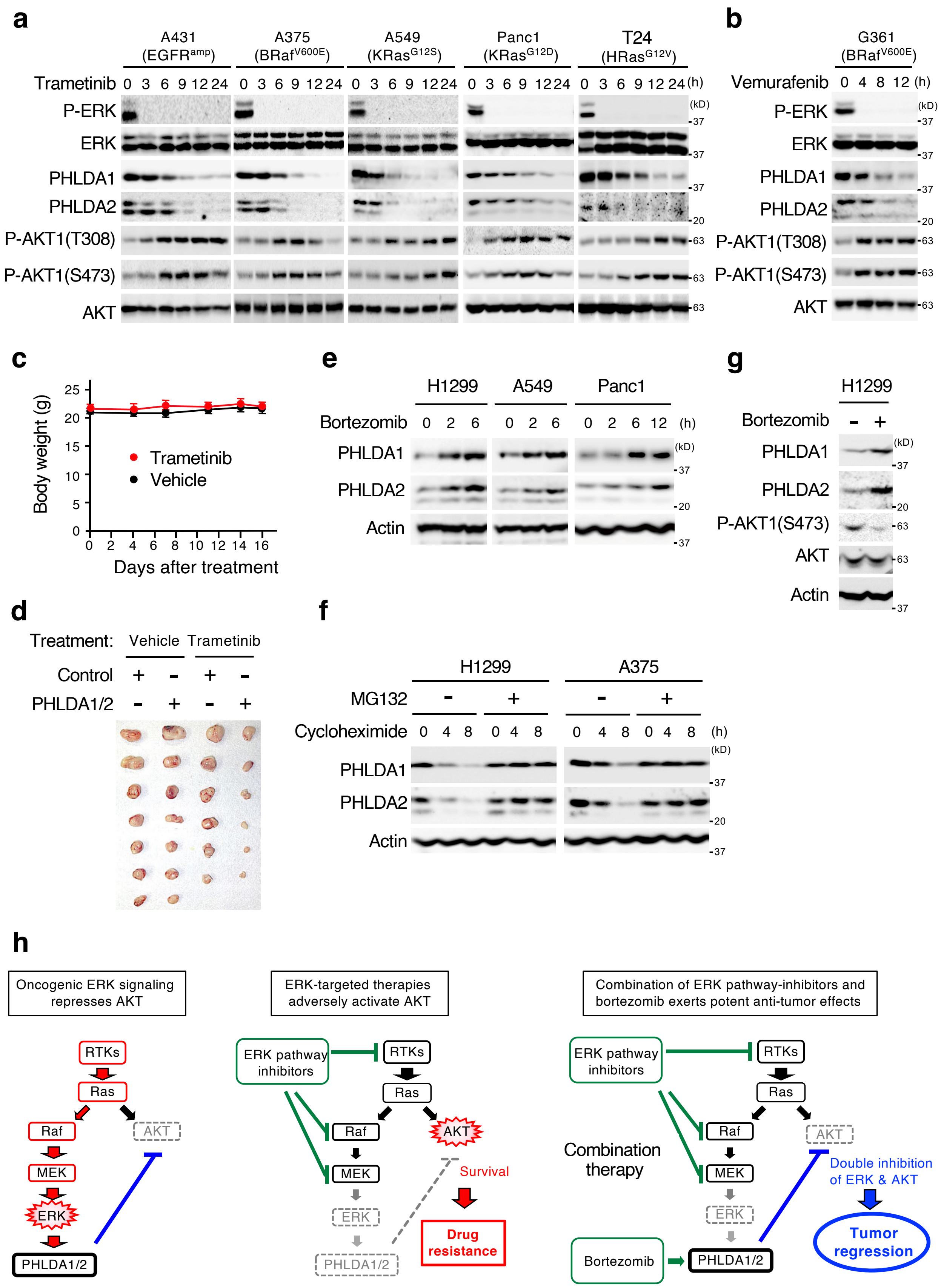

a
A431 (EGFRamp)
A375 (BRafV600E)
A549 (KRasG12S)
Panc1 (KRasG12D)
T24 (HRasG12V)
Trametinib 0 3 6 9 12 24 (h)
P-ERK
ERK
PHLDA1
PHLDA2
P-AKT1(T308)
P-AKT1(S473)
AKT
(kD)
37
20
63
b
G361 (BRafV600E)
Vemurafenib 0 4 8 12 (h)
c
Body weight (g)
Trametinib
Vehicle
Days after treatment
d
Treatment: Vehicle Trametinib
Control + - + -
PHLDA1/2 - + - +
e
H1299
A549
Panc1
Bortezomib 0 2 6 12 (h)
Actin
f
MG132 - +
Cycloheximide 0 4 8 0 4 8 (h)
g
Bortezomib - +
h
Oncogenic ERK signaling represses AKT
ERK-targeted therapies adversely activate AKT
Combination of ERK pathway-inhibitors and bortezomib exerts potent anti-tumor effects
RTKs
Ras
Raf
MEK
ERK
AKT
PHLDA1/2
ERK pathway inhibitors
Survival
Drug resistance
Combination therapy
Bortezomib
Double inhibition of ERK & AKT
Tumor regression

Supplementary Fig. 9 PHLDA1/2 downregulation by ERK pathway inhibitors induces AKT activation in cancer cells, thereby conferring resistance to the anti-cancer drugs.

a, **b**, Inhibition of aberrant ERK activity in cancer cells activates AKT through the downregulation of PHLDA1/2. The indicated human cancer cells harbouring various ERK-activating oncogenes were treated with 10 μM trametinib (**a**) or 10 μM vemurafenib (**b**) for the indicated times. The cell lysates were probed for the indicated proteins by immunoblotting.

c, **d**, Prevention of trametinib-induced PHLDA1/2 downregulation greatly enhances its anti-tumor efficacy in a mouse xenograft model. Nude mice bearing control H1299 and H1299-PHLDA1/2 tumors in the left and right flanks, respectively, were treated with vehicle or trametinib for 16 days. **c**, All mice were weighed twice a week following the first administration of study treatment, and no significant differences in weight were observed between vehicle- and trametinib-treated mice. Error bars, SEM (n=7 for vehicle-treated mice and n=6 for trametinib-treated mice). **d**, Photographs of xenograft tumors excised from individual mice after 16 days of treatment.

e, **g**, Bortezomib upregulates PHLDA1/2 expression. The indicated cancer cell lines were treated with 10 μM bortezomib for the indicated times. The cell lysates were probed for PHLDA1/2. Actin served as a loading control.

f, Proteasome inhibitors stabilize PHLDA1/2 proteins. H1299 and A375 cells were pretreated with 10 μM MG132 or were left untreated (-). After 30 min, the cells were treated with the protein synthesis inhibitor, cycloheximide (25 μg/ml), for the indicated times. PHLDA1/2 expression levels were assessed by immunoblotting as in **e**.

h, Model of the PHLDA1/2-mediated aberrant signaling crosstalk between ERK and AKT in cancer, and of how its disruption by bortezomib enhances the anti-tumor efficacy of ERK pathway-targeted therapies. (Left) Oncogene-induced hyperactive ERK signaling in cancer suppresses pro-survival AKT signaling through the induction of PHLDA1/2 expression. (Middle) Treatment of tumors with ERK pathway-targeted drugs downregulates PHLDA1/2 expression and leads to activation of AKT signaling in cancer cells, thereby conferring resistance to the drugs. (Right) Combination treatment of ERK pathway inhibitors and bortezomib can counteract ERK inhibitor-mediated downregulation of PHLDA1/2 expression and the resulting high AKT activity in cancer cells, thereby exerting potent anti-tumor effects with the result that cancer cells readily undergo apoptotic cell death.

Source data are provided as a Source Data file.
