# Supplementary material for: Qualitative differences in disease-associated MEK mutants reveal molecular signatures and aberrant signaling-crosstalk in cancer
Source: Nat Commun. 2022 Jul 13;13:4063. doi: 10.1038/s41467-022-31690-w (PMC9279491; doi:10.1038/s41467-022-31690-w)
Supplement: Supplementary file 2 — Description of Additional Supplementary Files [file 41467_2022_31690_MOESM2_ESM.pdf]

File name: Supplementary Movies 1-3

Description: Time-lapse imaging of the nuclear translocation of ERK. HEK293 cells stably expressing either HA-MEK1 (Supplementary movie 1), HA-MEK1(F53S) (Supplementary movie 2) or HA-MEK1(K57N) (Supplementary movie 3) were stimulated with EGF (5 ng/ml). The localization of ERK1-GFP was monitored with a time-lapse microscopy system every 90 sec over a period of 30 min. The mean percentage of nuclear-localized ERK during EGF stimulation was plotted and is shown in Fig. 4d.
