# Supplementary material for: Qualitative differences in disease-associated MEK mutants reveal molecular signatures and aberrant signaling-crosstalk in cancer
Source: Nat Commun. 2022 Jul 13;13:4063. doi: 10.1038/s41467-022-31690-w (PMC9279491; doi:10.1038/s41467-022-31690-w)
Supplement: Supplementary file 6 — Reporting Summary [file 41467_2022_31690_MOESM6_ESM.pdf]

nature portfolio

Corresponding author(s): Mutsuhiro Takekawa

Last updated by author(s): 2022-7-4

Reporting Summary

Nature Portfolio wishes to improve the reproducibility of the work that we publish. This form provides structure for consistency and transparency in reporting. For further information on Nature Portfolio policies, see our Editorial Policies and the Editorial Policy Checklist.

Statistics

For all statistical analyses, confirm that the following items are present in the figure legend, table legend, main text, or Methods section.

n/a	Confirmed	
☐	☒	The exact sample size (*n*) for each experimental group/condition, given as a discrete number and unit of measurement
☐	☒	A statement on whether measurements were taken from distinct samples or whether the same sample was measured repeatedly
☐	☒	The statistical test(s) used AND whether they are one- or two-sided<br>*Only common tests should be described solely by name; describe more complex techniques in the Methods section.*
☒	☐	A description of all covariates tested
☐	☒	A description of any assumptions or corrections, such as tests of normality and adjustment for multiple comparisons
☐	☒	A full description of the statistical parameters including central tendency (e.g. means) or other basic estimates (e.g. regression coefficient) AND variation (e.g. standard deviation) or associated estimates of uncertainty (e.g. confidence intervals)
☐	☒	For null hypothesis testing, the test statistic (e.g. *F*, *t*, *r*) with confidence intervals, effect sizes, degrees of freedom and *P* value noted<br>*Give P values as exact values whenever suitable.*
☒	☐	For Bayesian analysis, information on the choice of priors and Markov chain Monte Carlo settings
☒	☐	For hierarchical and complex designs, identification of the appropriate level for tests and full reporting of outcomes
☒	☐	Estimates of effect sizes (e.g. Cohen's *d*, Pearson's *r*), indicating how they were calculated

Our web collection on statistics for biologists contains articles on many of the points above.

Software and code

Policy information about availability of computer code

Data collection	Immunoblotting data were collected using the LAS-1000 Plus or ImageQuant LAS 4000 (Fujifilm).<br>qRT-PCR data were collected using TaKaRa Real Time PCR system.
Data analysis	GraphPad Prism 5.0, GSEA (ver. 4.1.0), XDS (ver. May 1, 2016), COOT (ver. 0.8.9), Phenix (ver. 1.11.1), CCP4 (ver. 6.5.004), PyMOL (ver. 2.1.0), and Image J (ver. 1.53a).

For manuscripts utilizing custom algorithms or software that are central to the research but not yet described in published literature, software must be made available to editors and reviewers. We strongly encourage code deposition in a community repository (e.g. GitHub). See the Nature Portfolio guidelines for submitting code & software for further information.

Data

Policy information about availability of data

All manuscripts must include a data availability statement. This statement should provide the following information, where applicable:

- Accession codes, unique identifiers, or web links for publicly available datasets
- A description of any restrictions on data availability
- For clinical datasets or third party data, please ensure that the statement adheres to our policy

The microarray data generated in this study has been deposited in the Gene Expression Omnibus (GEO) database under accession code GSE165823 [https://www.ncbi.nlm.nih.gov/geo/query/acc.cgi?acc=GSE165823]. The crystal structure of MEK1(C121S) reported in this paper has been deposited in the Protein Data Bank (PDB, ID code 7F2X) [http://doi.org/10.2210/pdb7F2X/pdb]. The structural data of wild-type MEK1 (PDB ID:, 3EQC, 3EQD, 3EQF, 3EQG, 3EQH, 3EQI, 3SLS, 3W8Q, 3ZLS, 3ZLX, 3ZLY, 3ZLW, 3ZM4, 5BX0, and 5HZE), MEK1-KSR2 complex (PDB ID: 2Y4I), MEK1-BRaf complex (PDB ID: 4MNE), and PKA (PDB ID: 1ATP and 4HPU) were obtained from the PDB database. All other data are available in the article and its Supplementary information/Source Data file, or from the corresponding

author upon reasonable request. Source data are provided with this paper.

Field-specific reporting

Please select the one below that is the best fit for your research. If you are not sure, read the appropriate sections before making your selection.

- [x] Life sciences
- [ ] Behavioural & social sciences
- [ ] Ecological, evolutionary & environmental sciences

For a reference copy of the document with all sections, see nature.com/documents/nr-reporting-summary-flat.pdf

Life sciences study design

All studies must disclose on these points even when the disclosure is negative.

Sample size	Sample sizes are indicated in the figure legends and/or listed within the figure panel. statistical analyses are described in "Methods".<br>No power analysis was used for sample sizes and replicates, but these were determined based on similar studies (e.g., ref.11 and ref.17)
Data exclusions	No data was excluded from analysis.
Replication	All experiments were repeated at least three times, giving similar results.
Randomization	Age and sex matched nude mice (BALB-c/nu) were allocated randomly into experimental groups. All other samples were randomly allocated into experimental groups.
Blinding	Investigators were not blinded during the experiments as all samples were processed and analyzed with the same protocol within an experiment. Therefore, prior knowledge had no impact on data output.

Reporting for specific materials, systems and methods

We require information from authors about some types of materials, experimental systems and methods used in many studies. Here, indicate whether each material, system or method listed is relevant to your study. If you are not sure if a list item applies to your research, read the appropriate section before selecting a response.

Materials & experimental systems

n/a	Involved in the study	
[ ]	[x]	Antibodies
[ ]	[x]	Eukaryotic cell lines
[x]	[ ]	Palaeontology and archaeology
[ ]	[x]	Animals and other organisms
[x]	[ ]	Human research participants
[x]	[ ]	Clinical data
[x]	[ ]	Dual use research of concern

Methods

n/a	Involved in the study	
[x]	[ ]	ChIP-seq
[x]	[ ]	Flow cytometry
[x]	[ ]	MRI-based neuroimaging

Antibodies

Antibodies used

For immunoblotting and immunofluorescent staining, the following primary antibodies were used at a dilution of 1:1000:

anti-HA F-7 (Santa Cruz Biotechnology, sc-7392)
anti-GST B-14 (Santa Cruz Biotechnology, sc-138)
anti–Myc 9E10 (Santa Cruz Biotechnology, sc-40)
anti-TFPI2 B-7 (Santa Cruz Biotechnology, sc-48380)
anti-MEK1 H-8 (Santa Cruz Biotechnology, sc-6250)
anti-ERK (Santa Cruz Biotechnology, sc-94)
anti-C-Raf-1 (Santa Cruz Biotechnology, sc-227)
anti-B-Raf (Santa Cruz Biotechnology, sc-166)
anti-HA 16B12 (Covance, MMS-101R)
anti-HA 3F10 (Roche, 11867423001)
anti-Flag M2 (Sigma-Aldrich, F1804)
anti-TM4SF19 (Sigma-Aldrich, SAB1102826)
anti-EMP1 (Sigma-Aldrich, SAB1302714)
anti-phospho-Raf-1 56A6 (Cell Signaling Technology, 9427)
anti-phospho-MEK1/2 41G9 (Cell Signaling Technology, 9154)
anti-phospho-AKT1(T308) 244F9 (Cell Signaling Technology, 4056)
anti-phospho-AKT1(S473) D9E (Cell Signaling Technology, 4060)

anti-phospho-rpS6 D57.2.2E (Cell Signaling Technology, 4858)
anti-rpS6 5G10 (Cell Signaling Technology, 2217)
anti-MEK2 13E3 (Cell Signaling Technology, 9147)
anti-GDF15 D2A3 (Cell Signaling Technology, 8479)
anti-phospho-ERK1/2 (Cell Signaling Technology, 9101)
anti-AKT (Cell Signaling Technology, 9272)
anti-cleaved caspase3 (Cell Signaling Technology, 9661)
anti-PARP (Cell Signaling Technology, 9542)
anti-PHLDA1 EPR6674 (Abcam, ab133654)
anti-PHLDA2 (Abcam, ab58379)
anti-PHLDA2 (Proteintech, 14661-1-AP)
anti-COL14A1 (Abcam, ab101464)
anti-TM4SF1 (Abcam, ab113504)
anti-β-Actin (FUJIFILM Wako, 010-27841)

The following secondary antibodies were used at a dilution of 1:5000 (anti-mouse HRP Ab), 1:2500 (anti-Rabbit HRP Ab), or 1:2000 (Alexa-Fluor anti-mouse IgG1):

anti-mouse HRP antibody (NA931, Cytiva)
anti-rabbit HRP antibody (NA934, Cytiva)
Alexa-Fluor 488 goat anti-mouse IgG1 (A-21121, Molecular Probe)

Validation

All antibodies used for western blotting were purchased from commercial vendors. Validation of antibodies used in current study is described in technical data sheets provided by manufacturers websites:

anti-HA F-7 (Santa Cruz Biotechnology, sc-7392) : https://www.scbt.com/ja/p/ha-probe-antibody-f-7
anti-GST B-14 (Santa Cruz Biotechnology, sc-138): https://www.scbt.com/p/gst-antibody-b-14?requestFrom=search
anti–Myc 9E10 (Santa Cruz Biotechnology, sc-40): https://www.scbt.com/p/c-myc-antibody-9e10?requestFrom=search
anti-TFPI2 B-7 (Santa Cruz Biotechnology, sc-48380): https://www.scbt.com/p/tfpi-2-antibody-b-7?requestFrom=search
anti-MEK1 H-8 (Santa Cruz Biotechnology, sc-6250): https://www.scbt.com/p/mek-1-antibody-h-8?requestFrom=search
anti-ERK (Santa Cruz Biotechnology, sc-94): https://www.scbt.com/p/erk-1-antibody-k-23?requestFrom=search
anti-C-Raf-1 (Santa Cruz Biotechnology, sc-227): https://www.scbt.com/p/raf-1-antibody-c-20?requestFrom=search
anti-B-Raf (Santa Cruz Biotechnology, sc-166): https://www.scbt.com/p/raf-b-antibody-c-19?requestFrom=search
anti-HA 16B12 (Covance, MMS-101R): https://www.biolegend.com/ja-jp/products/anti-ha-11-epitope-tag-antibody-11071
anti-HA 3F10 (Roche, 11867423001): https://www.sigmaaldrich.com/US/en/product/roche/roahaha?gclid=CjwKCAjwv-GUBhAzEiwASUMm4jbLh8W6TgpVxjx4EM5jcF6FhtOwfDSHFwBvzs2IKB9T7pgARtBqSBoCMAAQAvD_BwE
anti-Flag M2 (Sigma-Aldrich, F1804): https://www.sigmaaldrich.com/US/en/product/sigma/f1804
anti-TM4SF19 (Sigma-Aldrich, SAB1102826): https://www.sigmaaldrich.com/US/en/product/sigma/sab1102826
anti-EMP1 (Sigma-Aldrich, SAB1302714): https://www.sigmaaldrich.com/US/en/product/sigma/sab1302714
anti-phospho-Raf-1 56A6 (Cell Signaling Technology, 9427): https://en.cellsignal.jp/products/primary-antibodies/phospho-c-raf-ser338-56a6-rabbit-mab/9427
anti-phospho-MEK1/2 41G9 (Cell Signaling Technology, 9154): https://www.cellsignal.jp/products/primary-antibodies/phospho-mek1-2-ser217-221-41g9-rabbit-mab/9154
anti-phospho-AKT1(T308) 244F9 (Cell Signaling Technology, 4056): https://www.cellsignal.jp/products/primary-antibodies/phospho-akt-thr308-244f9-rabbit-mab/4056
anti-phospho-AKT1(S473) D9E (Cell Signaling Technology, 4060): https://www.cellsignal.jp/products/primary-antibodies/phospho-akt-ser473-d9e-xp-rabbit-mab/4060
anti-phospho-rpS6 D57.2.2E (Cell Signaling Technology, 4858): https://www.cellsignal.jp/products/primary-antibodies/phospho-s6-ribosomal-protein-ser235-236-d57-2-2e-xp-rabbit-mab/4858
anti-rpS6 5G10 (Cell Signaling Technology, 2217): https://www.cellsignal.jp/products/primary-antibodies/s6-ribosomal-protein-5g10-rabbit-mab/2217
anti-MEK2 13E3 (Cell Signaling Technology, 9147): https://www.cellsignal.jp/products/primary-antibodies/mek2-13e3-rabbit-mab/9147anti-GDF15 D2A3 (Cell Signaling Technology, 8479)
https://www.cellsignal.jp/products/primary-antibodies/mic-1-d2a3-rabbit-mab/8479anti-phospho-ERK1/2 (Cell Signaling Technology, 9101)
anti-AKT (Cell Signaling Technology, 9272): https://www.cellsignal.jp/products/primary-antibodies/akt-antibody/9272
anti-cleaved caspase3 (Cell Signaling Technology, 9661)
https://www.cellsignal.jp/products/primary-antibodies/cleaved-caspase-3-asp175-antibody/9661
anti-PARP (Cell Signaling Technology, 9542): https://www.cellsignal.jp/products/primary-antibodies/parp-antibody/9542
anti-PHLDA1 EPR6674 (Abcam, ab133654): https://www.abcam.co.jp/phlda1-antibody-epr6674-ab133654.html
anti-PHLDA2 (Abcam, ab58379): https://www.abcam.co.jp/tssc3-antibody-ab58379.html
anti-PHLDA2 (Proteintech, 14661-1-AP): https://www.ptglab.co.jp/products/PHLDA2-Antibody-14661-1-AP.htm
anti-COL14A1 (Abcam, ab101464): https://www.abcam.co.jp/col14a1-antibody-ab101464.html
anti-TM4SF1 (Abcam, ab113504): https://www.abcam.co.jp/transmembrane-4-l6-family-member-1-antibody-ab113504.html
anti-β-Actin (FUJIFILM Wako, 010-27841): https://labchem-wako.fujifilm.com/us/product/detail/W01W0101-2784.html
Alexa-Fluor 488 goat anti-mouse IgG1 (A-21121, Molecular Probe): https://www.thermofisher.com/antibody/product/Goat-anti-Mouse-IgG1-Cross-Adsorbed-Secondary-Antibody-Polyclonal/A-21121
Anti-mouse HRP antibody (NA931, Cytiva): https://www.sigmaaldrich.com/US/en/product/sigma/gena9311ml

Anti-rabbit HRP antibody (NA934, Cytiva): https://www.sigmaaldrich.com/US/en/product/sigma/gena9341ml

Eukaryotic cell lines

Policy information about cell lines

Cell line source(s)	A375 (CRL-1619), Sk-Mel28 (HTB-72), H1299 (CRL-5803), and HT29 (HTB-38) were obtained from ATCC. HEK293 (RCB1637), G361 (RCB0991), A549 (RCB3677), T24 (RCB2536), A431 (RCB0202), Panc1 (RCB2095), HCT116 (RCB2979), and GP2-293 (RCB2354) were obtained from RIKEN cell bank. WiDr (JCRB0224) was obtained from Japanese Cancer Research Resources Bank (JCRB). Plat-E cells and MEK1-/- MEFs were originally generated and provided by T. Kitamura (University of Tokyo) and by J. Charron (Université Laval, Québec), respectively.
Authentication	All cell lines used were obtained as pre-authenticated lines from ATCC, RIKEN cell bank, or JCRB. We did not further authenticate the cell lines.
Mycoplasma contamination	All cells used in this manuscript were routinely tested for mycoplasma contamination using a PCR-based method, and verified as mycoplasma-negative.
Commonly misidentified lines (See ICLAC register)	WiDr (ICLAC-00103) have been reported to be derived from HT29 and noted on the Cell Model Passport (https://cellmodelpassports.sanger.ac.uk) and JCRB (https://cellbank.nibiohn.go.jp/~cellbank/en/search_res_det.cgi?ID=308#), but they were included in the analysis for diversity owing to their oncogene status (BRaf-V600E) and tissue origin (adenocarcinoma).

Animals and other organisms

Policy information about studies involving animals; ARRIVE guidelines recommended for reporting animal research

Laboratory animals	Five-weeks-old female nude mice (BALB-c/nu) were purchased from Oriental Yeast (Tokyo, Japan) and bred in The Laboratory Animal Research Center (LARC) of The Institute of Medical Science, The University of Tokyo (IMSUT). Housing conditions: temperature 22 ± 2°C, humidity 55 ± 5%, light/dark cycle 12 hour/12 hour (8 am - 20 pm light).
Wild animals	The study did not involve wild animals, no animals in the study were collected from the field.
Field-collected samples	The study did not involve wild animals, no animals in the study were collected from the field.
Ethics oversight	The animal experiment in this study was approved by the animal experiment committee at the Institute of Medical Science, The University of Tokyo (IMSUT) (approval number: A16-5), and animal care was conducted in accordance with institutional guidelines.

Note that full information on the approval of the study protocol must also be provided in the manuscript.
